# LncOCMRL1 promotes oral squamous cell carcinoma growth and metastasis via the RRM2/EMT pathway

**DOI:** 10.1186/s13046-024-03190-w

**Published:** 2024-09-30

**Authors:** Nan Lu, Qiming Jiang, Tianshu Xu, Qiyuan Gao, Yuepeng Wang, Zixian Huang, Zhiquan Huang, Xiaoding Xu

**Affiliations:** 1 Guangdong Provincial Key Laboratory of Malignant Tumor Epigenetics and Gene Regulation, Guangdong-Hong Kong Joint Laboratory for RNA Medicine, Medical Research Center, Sun Yat-Sen Memorial Hospital, Sun Yat-Sen University, Guangzhou, 510120 PR China; 2 Guangzhou Key Laboratory of Medical Nanomaterials, Sun Yat-Sen Memorial Hospital, Sun Yat-Sen University, Guangzhou, 510120 PR China; 3 Nanhai Translational Innovation Center of Precision Immunology, Sun Yat-Sen Memorial Hospital, Foshan, 528200 PR China; 4 Department of Oral and Maxillofacial Surgery, Sun Yat-sen Memorial Hospital, Sun Yat-sen University, Guangzhou, Guangdong 510120 PR China; 5 Department of Prosthodontics and Implantology, Shenzhen University Affiliated Shenzhen Stomatology Hospital, Shenzhen, 518001 PR China; 6 Department of Pharmacology, Joint Laboratory of Guangdong-Hong Kong Universities for Vascular Homeostasis and Diseases, School of Medicine, Southern University of Science and Technology, Shenzhen, 518001 PR China

**Keywords:** OSCC metastasis, lncOCMRL1, EMT, Nanoparticles, siRNA delivery

## Abstract

**Background:**

Long noncoding RNAs (lncRNAs) are widely involved in cancer development and progression, but the functions of most lncRNAs have not yet been elucidated. Metastasis is the main factor restricting the therapeutic outcomes of various cancer types, including oral squamous cell carcinoma (OSCC). Therefore, exploring the key lncRNAs that regulate OSCC metastasis and elucidating their molecular mechanisms will facilitate the development of new strategies for effective OSCC therapy.

**Methods:**

We analyzed the lncRNA expression profiles of tumor tissues from OSCC patients with and without cervical lymph node metastasis, and OSCC cell lines. We revealed high expression of oral squamous cell carcinoma metastasis-related lncRNA 1 (lncOCMRL1) in OSCC patient tumor tissues with lymph node metastasis and highly metastatic OSCC cell lines. The effects of lncOCMRL1 knockdown on the invasion, migration and proliferation abilities of OSCC cells were explored through qRT-PCR, Transwell, colony formation, and cell proliferation experiments. The mechanism by which lncOCMRL1 promotes OSCC metastasis and proliferation was explored through RNA pull-down, silver staining, mass spectrometry, RIP, and WB experiments. To increase its translational potential, we developed a reduction-responsive nanodelivery system to deliver siRNA for antitumor therapy.

**Results:**

We determined that lncOCMRL1 is highly expressed in OSCC metastatic tumor tissues and cells. Functional studies have shown that high lncOCMRL1 expression can promote the growth and metastasis of OSCC cells both in vivo and in vitro. Mechanistically, lncOCMRL1 could induce epithelial-mesenchymal transition (EMT) via the suppression of RRM2 ubiquitination and thereby promote the proliferation, invasion, and migration of OSCC cells. We further constructed reduction-responsive nanoparticles (NPs) for the systemic delivery of siRNAs targeting lncOCMRL1 and demonstrated their high efficacy in silencing lncOCMRL1 expression in vivo and significantly inhibited OSCC tumor growth and metastasis.

**Conclusions:**

Our results suggest that lncOCMRL1 is a reliable target for blocking lymph node metastasis in OSCC.

**Supplementary Information:**

The online version contains supplementary material available at 10.1186/s13046-024-03190-w.

## Background

Head and neck cancer (HNC) is the sixth most common cancer worldwide and primarily affects the oral cavity, lip, oropharynx, hypopharynx, and larynx. Oral squamous cell carcinoma (OSCC) arises primarily from the oral mucosal epithelium and accounts for approximately 90% of oral cancer cases [1–3]. Clinical statistics indicate that most OSCC patients develop local tumor invasion and lymph node metastasis, and the five-year survival rate is only 50% [4, 5]. Lymph node metastasis is considered an important reason for the poor prognosis of OSCC patients [6, 7].

Long noncoding RNAs (lncRNAs) are noncoding RNAs with a length of more than 200 nucleotides, and abnormal expression and mutation of lncRNAs are closely related to tumor metastasis [8–11]. HOTAIR and NEAT1 are overexpressed in tumors such as breast cancer, hepatocellular carcinoma, and non-small cell lung cancer. They are currently the most studied lncRNAs; they promote tumor invasion and metastasis and serve as markers for the diagnosis and prognosis prediction of various cancers [12–14]. In OSCC, few lncRNAs are related to tumor metastasis, and the exploration of additional unknown lncRNAs could provide a theoretical basis for the diagnosis and treatment of OSCC [14, 15].

To explore the key lncRNAs contributing to OSCC metastasis and investigate their potential application, we compared the lncRNA expression profiles of tumor tissues from OSCC patients with and without cervical lymph node metastasis and OSCC cells and demonstrated the upregulation of oral cancer metastasis-related lncRNA 1 (lncOCMRL1) in metastatic tumor tissues and highly metastatic cells. A mechanistic study indicated that high lncOCMRL1 expression could bind to the oncogenic protein RRM2, inhibit its ubiquitination, maintain protein stability, promote the EMT phenotype of OSCC cells, and increase their invasion and migration capabilities. Considering the difficulty of regulating lncRNA expression in vivo, we developed a reduction-responsive nanoplatform for the systemic delivery of lncOCMRL1 siRNA (siOCMRL1). The in vivo results revealed that this siRNA delivery system could efficiently transport siOCMRL1 into tumor tissues and significantly silence lncOCMRL1 expression, thereby leading to significant inhibition of tumor growth and metastasis in both OSCC tongue orthotopic and patient-derived xenograft (PDX) tumor models. (Scheme 1).

**Schme. 1:**
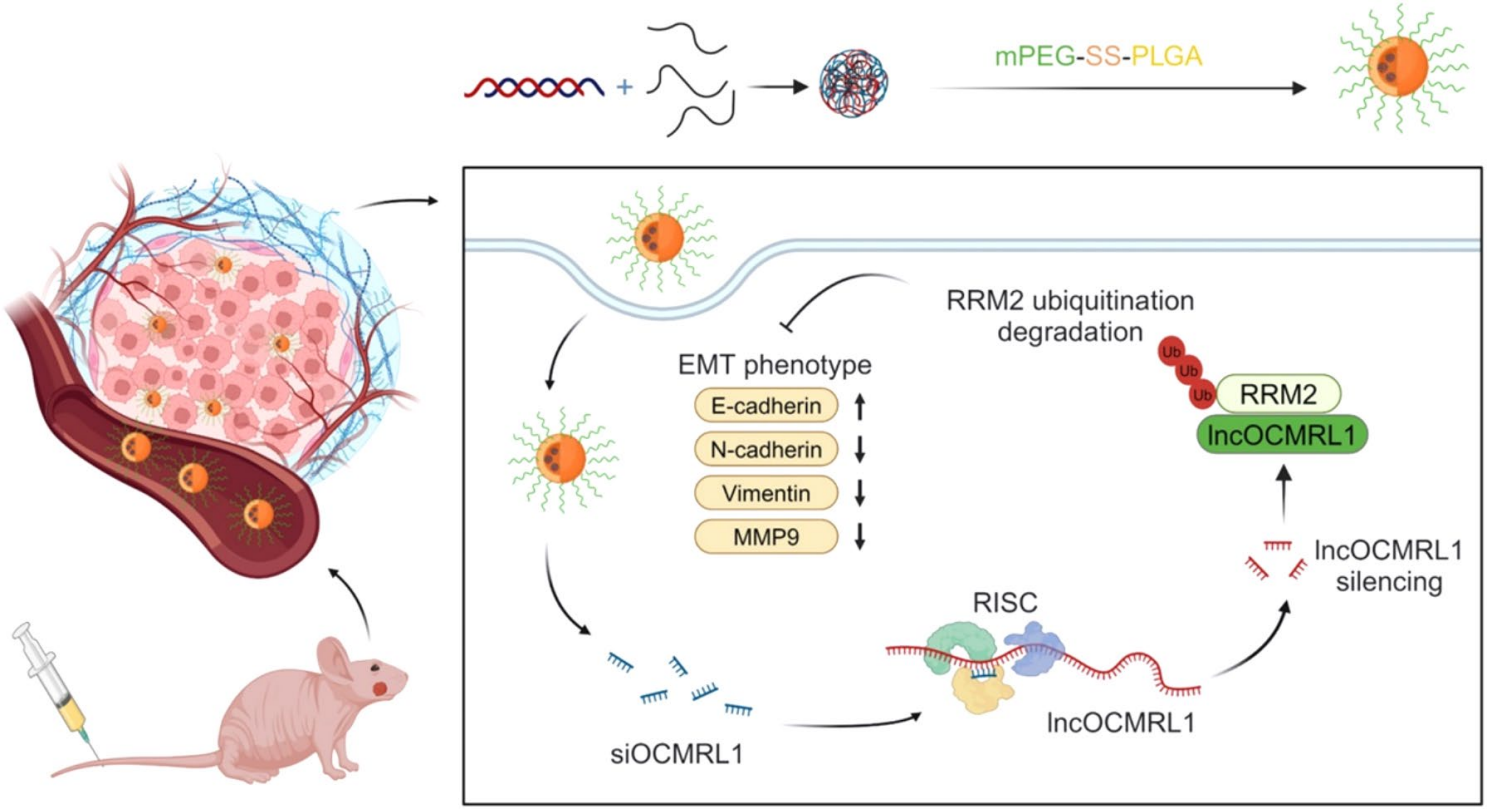
Schematic working model of the mechanism by which high lncOCMRL1 expression promotes RRM2 stability and EMT to promote OSCC metastasis. LncOCMRL1, which is upregulated in OSCC, can bind to RRM2, suppress the ubiquitination of RRM2, and promote EMT in OSCC cells, ultimately promoting OSCC metastasis through the lncOCMRL1/RRM2/EMT axis

## Methods

### Patient and tissue samples

The tissue samples used in this study were all from patients in the Department of Oral and Maxillofacial Surgery, Sun Yat-sen Memorial Hospital, Sun Yat-sen University. All patient tissue samples were collected in accordance with the International Ethical Guidelines for Biomedical Research Involving Human Subjects (CIOMS) and with informed consent from the donors. The study was approved by the Institutional Review Board (IRB) of Sun Yat-Sen Memorial Hospital.

### RNA sequencing

Total RNA was extracted from tumor samples (tumor samples from OSCC patients without metastasis or with metastasis after surgery) and from CAL-27 and SCC-9 tumor cells via TRIzol. The quality of the collected RNA was assessed, and the transcripts in the samples were analyzed via an Illumina analyzer. Sequencing and bioinformatics analysis were performed by IGE Biotechnology (Guangzhou, China).

### Cells and cell culture

In this study, four OSCC cell lines (SCC-9, CAL-27, HSC-6, and TCA-8113) and one tool cell line, human embryonic kidney (HEK 293T), were used. The SCC-9 cells were cultured in DMEM/F-12 supplemented with 10% FBS. CAL-27, HSC-6, TCA-8113 and HEK293T cells were cultured in DMEM supplemented with 10% FBS. All the cells were cultured in a cell incubator at a constant temperature of 37 °C containing 5% CO_2_.

### LncOCMRL1 silencing

SCC-9 and HSC-6 cells in 6-well plates (50,000 cells per well) were incubated with the NPs(siNC), NPs(si1), NPs(si2), Lipo3k/siNC, or Lipo3k/siOCMRL1complexes at a 50 nmol/L siRNA. Forty-eight hours later, the cells were trypsinized and collected for reverse transcription quantitative polymerase chain reaction (qRT-PCR), Western blot analysis and functional experiments. siRNAs were designed and produced by IGE Biotechnology (Guangzhou, China). The sequences of the siRNAs used were as follows: siNC, 5’-UUGGUGUCCUAUUACUGGGTT-3’; siOCMRL1-1, 5’-GCUAGAUUCUCGAAAGACA-3’; siOCMRL1-2, 5’-GGGUUAGUGUGCCUGACAA-3’; siRRM2-1, CCAUCGAGUACCAUGAUAUTT; and siRRM2-2, GGAGCGAUUUAGCCAAGAATT; siPVT1-1: GAGCUGCGAGCAAAGAUGUTT; and siPVT1-2: ACUUUAAGUGGAGGCUGAAUCAUCU; siLINC00662-1: GCUGCUGCCACUGUAAUAATT; and siLINC00662-1: GCAGGCGUACAACUAACAATT. Lipofectamine 3000 reagent (Invitrogen) was used to transfect the cells with siRNA.

### Cell migration and invasion

Twenty-four-well plates with 8 μm pore sizes of polycarbonate filters (Corning, 3422) coated with or without Matrigel were used. SCC-9 and HSC-6 cells (30,000 cells per well) were cultured in FBS-free DMEM/F-12 and DMEM in the upper layer of the chamber, and 700 µl of medium containing 10% FBS was added to the lower layer. After incubation for 24–48 h, the cells were fixed with 4% paraformaldehyde for 15 min and stained with 0.1% crystal violet for 10 min. Observations and imaging were performed under an Olympus optical electron microscope.

### Clony formation and proliferation

The cells were cultured in a 6-well plate at a density of 1,000 cells per well for 7–14 days to form cell colonies. The cells were fixed with 4% paraformaldehyde for 15 min, stained with 0.1% crystal violet for 10 min, and observed under an Olympus optical electron microscope. The cells were cultured in 96-well plates at a density of 1000 cells per well. After the cells were cultured for 24 h, the CCK-8 reagent was diluted with DMEM, 100 µL of the mixture was added to each well, and the cells were incubated at 37 °C for 2 h. A microplate reader was used to measure the optical density at a wavelength of 450 nm, after which statistical analysis of the data was performed.

### Construction of stably transfected cells

The target plasmid and packaging plasmid (pMD2G and psPAX2) were transfected into HEK 293T cells via Lipofectamine 3000 and P3000 (Invitrogen) at a ratio of 1.7:0.5:1. After 48 h, the virus-containing supernatant was collected and filtered through a 0.22 μm filter. The target cells were infected, and the stably transfected cells were screened. Successful transfection was verified by cell fluorescence imaging and qRT-PCR. Cells stably expressing HSC-6-luci, HSC-6-luci-shNC, HSC-6-luci-shOCMRL1, CAL-27-lncOCMRL1 and TCA-8113-lncOCMRL1 were constructed.

### Construction of an animal model of OSCC tongue orthotopic xenograft model using cells with stable knockdown of lncOCMRL1

The successfully constructed HSC-6-luci-shNC and HSC-6-luci-shOCMRL1 cells (5 × 10^5^/50 µl) were injected into the tongues of BALB/c-nu mice. One week after the injection, the tumors formed and were observed via an in vivo imaging system to confirm tumor formation. Tumor metastasis and growth were observed every week. In the third week, the tumors metastasized, and the shNC-treated mice exhibited a significant decrease in weight. The experiment was terminated, and in situ tumors of the tongue were harvested. Throughout the experiment, the weights of the mice were recorded every two days, and the tumor size and extent of tumor lymph node metastasis were also determined.

### RNA pull-down

The total protein from the HSC-6 cells was combined with the lncOCMRL1 RNA probe containing a tRSA scaffold at one end (streptavidin can recognize the tRSA structure) and then combined with streptavidin magnetic beads to form a complex. After SDS-PAGE, silver staining and mass spectrometry, the proteins that specifically bound to lncOCMRL1 were detected [16]. First, cell protein lysates were prepared by lysing cells in NP40 lysis buffer (containing 1 mM DTT with protease inhibitors and RNase inhibitors) to obtain reactive proteins. RNA probes were subsequently prepared, and the tRSA vector was used as a negative control; RNA was synthesized via the HyperScribeTM T7 High Yield Transcription Kit (APExBIO, K1047). Fifty pmol of RNA was denatured at 85 °C for 5 min and then cooled to 4 °C every 30 s to allow RNA folding. Two hundred micrograms of protein lysate was incubated with 50 pmol of denatured RNA for 2 h at 4 °C and then incubated with 30 µl of washed Dynabeads MyOne Streptavidin T1 (Invitrogen) magnetic beads overnight. After the beads were washed 5 times with NP40, the proteins were denatured by heating and separated via SDS-PAGE. Silver staining revealed differential bands, and mass spectrometry analysis was performed.

### RNA immunoprecipitation (RIP)

To obtain cell lysates, the cells were treated with 0.3% formaldehyde for intracellular cross-linking, 0.125 M glycine was added to stop the cross-linking reaction, and the cells were resuspended in 1 ml of NP40 lysis buffer (containing protease inhibitors and RNase inhibitors) and incubated with RRM2 antibody or IgG antibody for 2 h at 4 °C. The washed magnetic beads were added to the mixture and incubated at 4 °C overnight. After the magnetic beads were washed and the samples were treated with proteinase K, phenol-chloroform was used to extract and purify the RNA, and qRT-PCR was used to assess the enrichment of lncOCMRL1.

### Preparation of the NPs

The mPEG-S-S-PLGA polymer was dissolved in N, N-dimethylformamide (DMF) to form a uniform solution at a concentration of 20 mg/ml. G0-C14 were dissolved in DMF to form a homogeneous solution of 5 mg/ml. Then, 1 nmol of siRNA was dissolved in 10 µl of DEPC-H_2_O. Next, 200 µL of polymer, 50 µL of GO-C14 and 10 µL of siRNA were mixed evenly, after which the mixture was slowly added to 5 ml of deionized water and rotated at a constant speed (1000 rpm). The NPs were prepared via the nanoprecipitation method. Deionized water containing the mixture was added to an ultrafiltration tube (15 ml system, 100 KDa cutoff), which was then centrifuged vigorously (2800 rpm) to remove free compounds and deionized water. The NPs were washed with deionized water 3 times and finally diluted with 1 ml of deionized water. The particle size of the NPs was determined via dynamic light scattering (DLS, Malvern, USA). Cy5-labeled siRNA was used to determine the efficiency of siRNA encapsulation into the nanomaterials, which was measured on a microplate reader.

### Construction of an OSCC tongue orthotopic xenograft model and antitumor treatment

HSC-6-luci cells (5 × 10^5^/50 µl) in medium containing Matrigel were inoculated into the tongues of 5-week-old female BALB/c nude mice, and tumor formation was assessed via an in vivo imaging system. After tumor formation was observed for 7–14 days, the mice were divided into 4 groups (*n* = 5) and treated with (i) PBS, (ii) naked siOCMRL1, (iii) NPs(siNC), or (iii) NPs(siOCMRL1). The sequences in the NPs(siOCMRL1) use the siOCMRL1-1 sequences in Sect. 4.4 above. The mice were injected with 1 nmol of siRNA every two days for a total of three treatments. The mouse body weight, tumor size and tumor lymph node metastasis status were observed. After treatment, tumor tissues were collected from the mice for lncOCMRL1, Ki67 and RRM2 staining, and lymph nodes were collected for HE staining to observe lymph node metastasis.

### Generation of PDX-bearing mice and antitumor treatment

Fresh tumor tissue from oral cancer patients was cut to a size of 20–30 mg, and the tissue was transplanted subcutaneously into the right lower backs of the mice. The tumor size was measured every week. After the treatment standard (tumor volume of 60 mm^3^) was reached, the same groups as those in the OSCC tongue orthotopic xenograft model were treated with the NPs. After treatment, mouse tumors were collected for histological staining and statistical analysis.

### Statistical analysis

Kaplan-Meier (KM) curves were used to compare OS among different patient groups. GraphPad Prism (version 8.0) was used for visualization, graphing, and statistical analysis. A t test was used to compare the differences in qRT-PCR, tumor xenograft model features, and cell behaviors (migration, invasion, proliferation, etc.) between the different groups. Quantitative data are expressed as the standard deviation (SD) unless otherwise stated. Significance is indicated (* *p* < 0.05; * * *p* < 0.01; * * * *p* < 0.001). All the experiments were repeated three times.

## Results

### LncOCMRL1 is highly expressed in OSCC patients with metastasis and high lncOCMRL1 expression predicts a poor prognosis

**Fig. 1:**
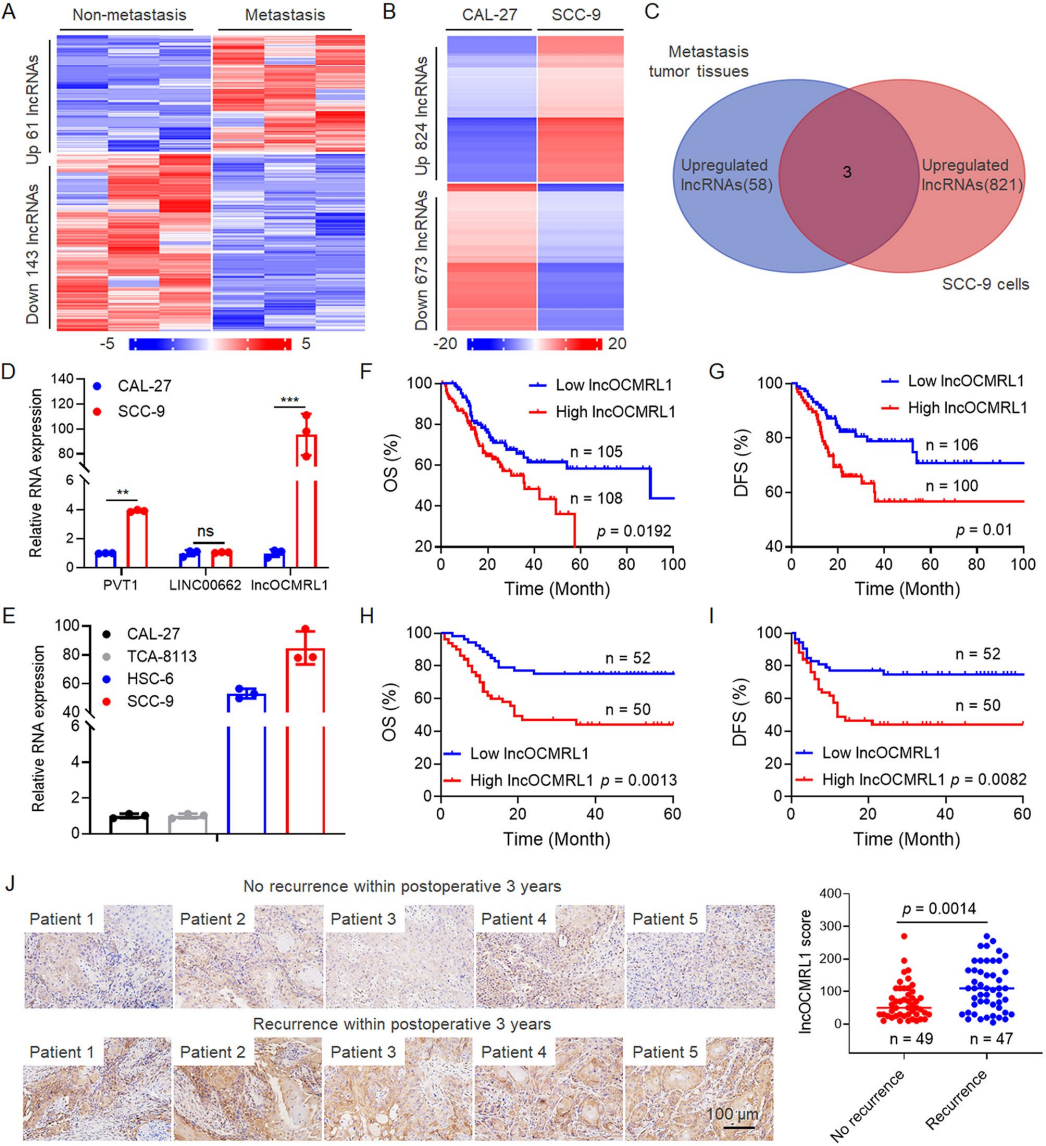
LncOCMRL1 is highly expressed in OSCC patients with tumor metastasis, indicating poor prognosis. (**A**) LncRNA expression profiles of tumor tissues from OSCC patients without cervical lymph node metastasis (*n* = 3) and with cervical lymph node metastasis (*n* = 3). The heatmap shows genes with log_2_FC > 3 and < -3. (**B**) LncRNA expression profiles of CAL-27 cells with low metastasis potential and SCC-9 cells with high metastasis potential. The heatmap shows genes with log_2_FC > 3 and < -3. (**C**) The number of overlapped lncRNAs upregulated in OSCC metastasis tumor tissues and SCC-9 cells. (**D**) Results of qRT-PCR analysis of the genes shown in (**C**) in SCC-9 and CAL-27 cells. (**E**) qRT-PCR analysis of lncOCMRL1 expression in four OSCC cell lines. (**F**-**G**) The TCGA database shows the OS (*n* = 213) (F) and DFS (*n* = 206) (**G**) of OSCC patients with different lncOCMRL1 expression levels.(**H**-**I**) OS (**H**) and DFS (**I**) of OSCC patients (*n* = 102) with different levels of lncOCMRL1 expression in their tumors. (**J**) ISH detection and statistical analysis of lncOCMRL1 expression in the tumor tissues of OSCC patients (*n* = 96) with postoperative recurrence and metastasis or without postoperative recurrence and metastasis (scale bar, 100 μm). The error bars represent the SDs of independent experiments. * *p* < 0.05; ** *p* < 0.01; *** *p* < 0.001

**Fig. 2:**
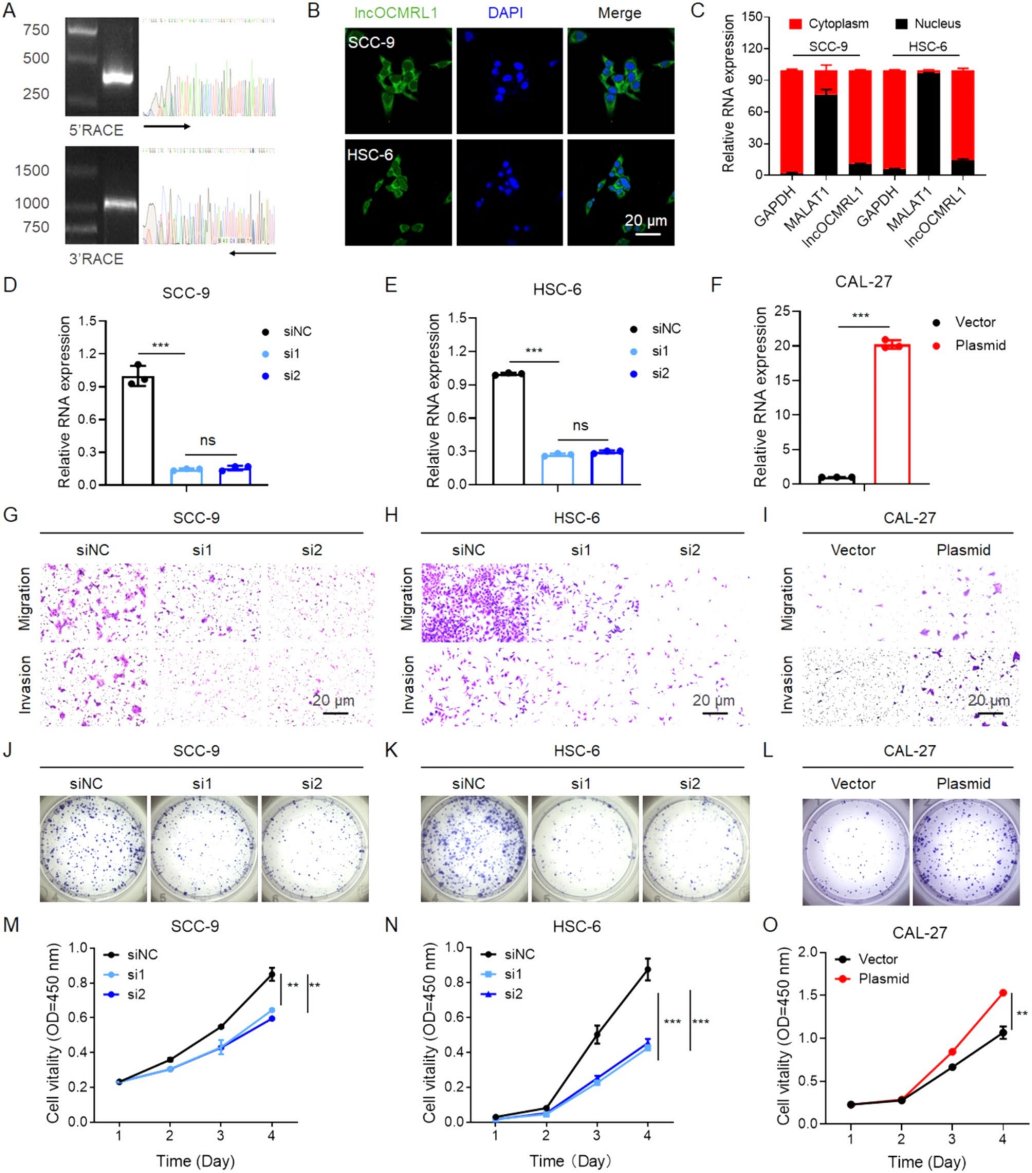
LncOCMRL1 is located mainly in the cytoplasm. LncOCMRL1 silencing inhibited OSCC cell migration, invasion and proliferation. (**A**) 5’ and 3’ RACE assays were performed to measure the full-length sequence of lncOCMRL1. Representative images showing 5’-RACE and 3’-RACE products separated by 1% agarose gel electrophoresis. (**B**) Assessment of lncOCMRL1 localization in SCC-9 and HSC-6 cells by FISH (scale bar, 100 μm). (**C**) The expression of lncOCMRL1 in SCC-9 and HSC-6 cells was determined by nuclear/cytoplasmic separation experiments. (**D**-**F**) Validation of lncOCMRL1 silencing in SCC-9 (**D**) and HSC-6 cells (**E**) treated with 50 nM siOCMRL1 by qRT-PCR and overexpression in CAL-27 cells by qRT-PCR (**F**). (**G**-**I**) Representative images of migration and invasion after lncOCMRL1 was silenced in SCC-9 (**G**) and HSC-6 cells (**H**) and after lncOCMRL1 was overexpressed in CAL-27 cells (**I**) (scale bar, 20 μm). (**J**-**L**) Representative images of the colony formation ability of SCC-9 (**J**) and HSC-6 cells (**K**) after lncOCMRL1 was silenced and after lncOCMRL1 was overexpressed in CAL-27 cells (**L**). (**M**-**O**) Proliferation of SCC-9 (**M**) and HSC-6 cells (**N**) with lncOCMRL1 silencing and after lncOCMRL1 was overexpressed in CAL-27 cells (**O**). The error bars represent the SDs of independent experiments. * *p* < 0.05; ** *p* < 0.01; *** *p* < 0.001

To identify the key lncRNAs that regulate OSCC metastasis, we collected tumor tissues from six OSCC patients with cervical lymph node metastasis (*n* = 3) and without metastasis (*n* = 3) and analyzed their lncRNA expression profiles via high-throughput sequencing technology (Table S1, Fig. 1A). We also evaluated the migration and invasion abilities of OSCC cells (Figure S1), and SCC-9 cells with highly metastatic characteristics and CAL-27 cells with low metastatic characteristics were selected for lncRNA sequencing (Fig. 1B). As shown in Fig. 1C, three lncRNAs were highly expressed in both the metastatic tissues of OSCC patients and highly metastatic SCC-9 cells (Fig. 1C). The expression of genes in OSCC cells was detected (Fig. 1D), and the results revealed that the expression of lncOCMRL1 was significantly increased in highly metastatic SCC-9 cells. We initially knocked down three candidate genes in SCC-9 and HSC-6 cells and explored their metastatic function and found that the migration and invasion abilities were significantly reduced after the expression of lncOCMRL1 was reduced (Figure S2, Fig. 2D-E and G-H). We detected the expression of lncOCMRL1 in OSCC cells and found that it was highly expressed in highly metastatic SCC-9 and HSC-6 cells (Fig. 1E). Therefore, lncOCMRL1 was selected as the main research target.

The Cancer Genome Atlas (TCGA) database revealed that lncOCMRL1 is highly expressed in the tumor tissues of OSCC patients and that the overall survival (OS, Fig. 1F) and disease-free survival (DFS, Fig. 1G) of patients are shortened. We analyzed the expression of lncOCMRL1 in tumor tissue samples from OSCC patients by in situ hybridization (ISH) and found that patients with high expression of lncOCMRL1 in tumor tissues had shortened OS (Fig. 1H) and DFS (Fig. 1I) times.We analyzed the expression of lncOCMRL1 in tumor tissue samples from OSCC patients without and with recurrence and metastasis via ISH and found that lncOCMRL1 was highly expressed in tumor tissue sections from patients with recurrence and metastasis (Fig. 1J). These results indicate that lncOCMRL1 may be involved in the OSCC metastasis process.

### High lncOCMRL1 expression promotes OSCC metastasis and proliferation

The full-length sequence of lncOCMRL1 was successfully obtained through rapid amplification of 5′ and 3′ cDNA ends (RACE) and was then used as the sequence for subsequent research (Fig. 2A, Supporting Information 1). We subsequently examined the subcellular localization of lncOCMRL1 via fluorescence in situ hybridization (FISH). We found that lncOCMRL1 was localized mainly in the cytoplasm in SCC-9 and HSC-6 cells (Fig. 2B), and consistent results were obtained via separation of the nuclear and cytoplasmic fractions (Fig. 2C). Therefore, we determined that lncOCMRL1 is a full-length 3979 bp lncRNA that is located mainly in the cytoplasm of OSCC cells. The transcript of lncOCMRL1 is ENST00000582008.1 (Ensembl database).

After basic information was obtained, we explored the function of lncOCMRL1. We used siOCMRL1 to knock down lncOCMRL1 in SCC-9 and HSC-6 cells, which exhibit high lncOCMRL1 expression (Fig. 2D-E). After lncOCMRL1 expression was downregulated, the migration and invasion abilities of OSCC cells were significantly inhibited (Fig. 2G-H). The cell proliferation ability was also analyzed. After lncOCMRL1 expression was knocked down, cell proliferation (Fig. 2M-N) and colony formation (Fig. 2J-K) were significantly inhibited. In contrast, lncOCMRL1 expression was upregulated in CAL-27 and TCA-8113 cells with low metastatic potential (Fig. 2F, Figure S3A), and cell migration and invasion were increased (Fig. 2I, Figure S3B). After lncOCMRL1 was overexpressed, cell proliferation (Fig. 2O, Figure S3D) and colony formation were significantly enhanced (Fig. 2L, Figure S3C). These results indicate that high lncOCMRL1 expression promotes OSCC development by regulating OSCC cell metastasis and proliferation.

**Fig. 3:**
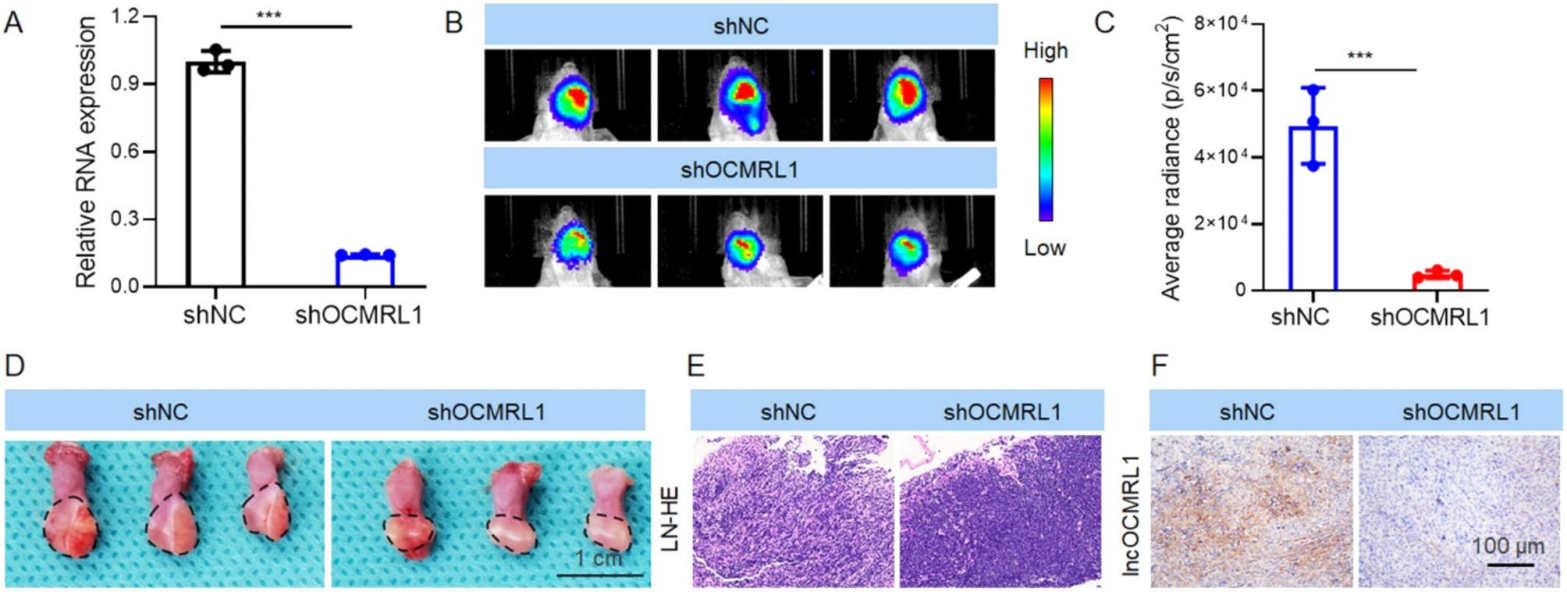
High lncOCMRL1 expression promotes OSCC growth and metastasis. (**A**) The successful generation of the HSC6-luci-shOCMRL1 cells was verified via qRT-PCR. (**B**) Bioluminescence image of mice after orthotopic injection of HSC-6-luci-shOCMRL1 cells. (**C**) Average tumor burden determined from the bioluminescence images shown in (**B**). (**D**) Images of orthotopic tumors in the tongue of mice after orthotopic injection of HSC-6-luci-shOCMRL1 cells into the tongue (scale bar, 1 cm) (**E**) HE staining showing lymph node metastasis in mice. (**F**) ISH was used to determine the stable silencing of lncOCMRL1 in tumors from mice (scale bar, 100 μm). The error bars represent the SDs of independent experiments. * *p* < 0.05; ** *p* < 0.01; *** *p* < 0.001

To clarify the effect of lncOCMRL1 on OSCC tumor metastasis and growth, we constructed HSC-6-luci cells with stable lncOCMRL1 knockdown, HSC-6-luci-shOCMRL1 (Fig. 3A). According to the OSCC tongue orthotopic xenograft model, compared with those in the control group, the tongue tumors in the BALB/c-nu mice in the shOCMRL1 group grew more slowly and metastasized less frequently to the cervical lymph nodes (Fig. 3B-F).

### High lncOCMRL1 expression suppresses RRM2 ubiquitination

**Fig. 4:**
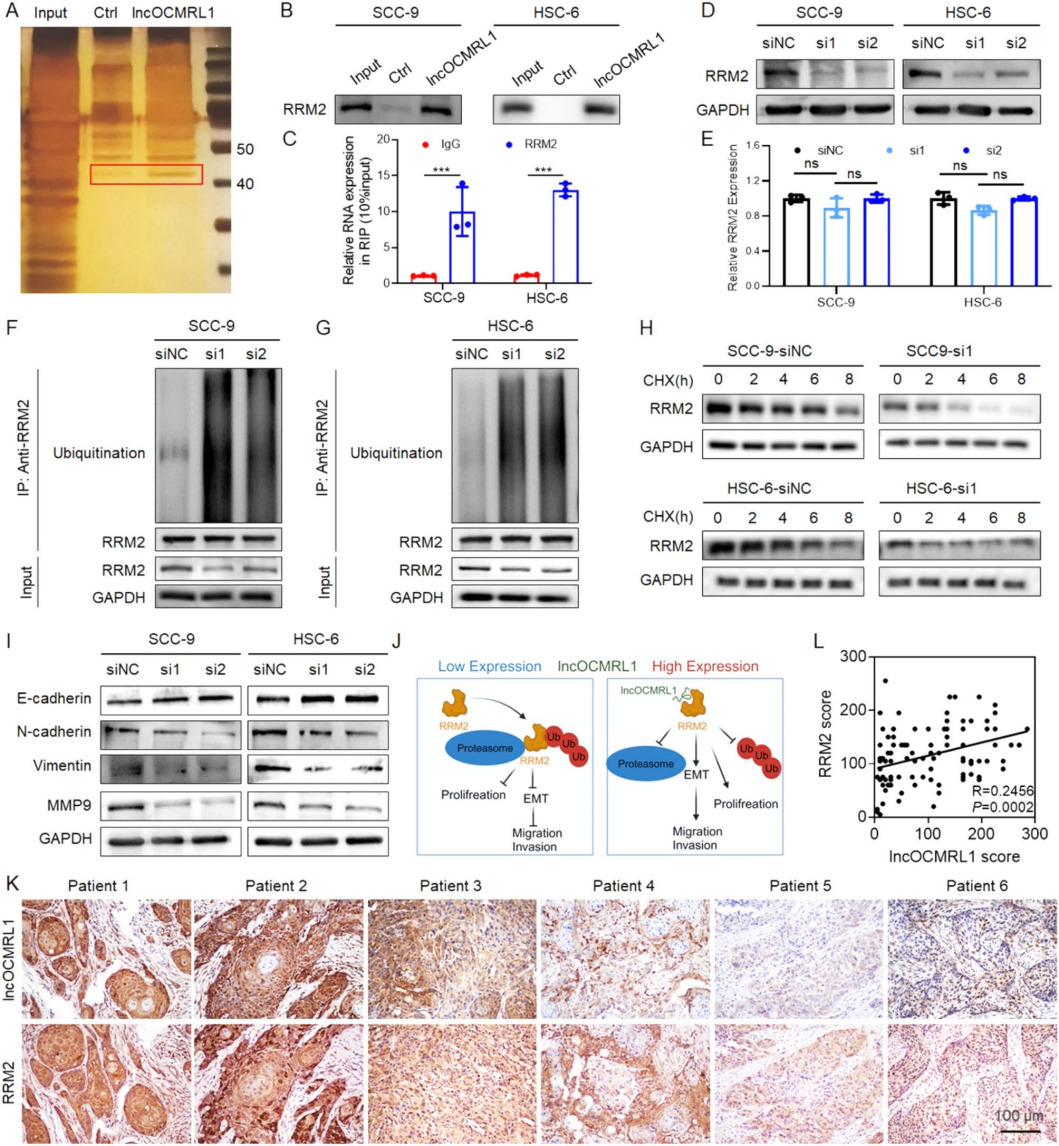
High lncOCMRL1 expression promotes OSCC cell migration, invasion and proliferation by inhibiting the ubiquitination and degradation of RRM2. (**A**) Silver staining of RNA-protein complexes determined by RNA pull down of lncOCMRL1 in HSC-6 cells. The band in the red box represents the protein specifically precipitated by lncOCMRL1. (**B**) Western blotting analysis of RNA pull-down in SCC-9 and HSC-6 cells. (**C**) In SCC-9 and HSC-6 cells, qRT-PCR analysis of RNA immunoprecipitation (RIP) for IgG and RRM2 revealed the mutual binding of lncOCMRL1 and RRM2. (**D**) In SCC-9 and HSC-6 cells, changes in RRM2 protein levels were analyzed by Western blotting after lncOCMRL1 silencing. (**E**) Analysis of changes in RRM2 mRNA expression after lncOCMRL1 silencing in SCC-9 and HSC-6 cells via qRT-PCR. (**F**-**G**) Western blotting analysis of lncOCMRL1 ubiquitination in MG132-treated SCC-9 (**F**) and HSC-6 cells (**G**) after lncOCMRL1 silencing. (**H**) SCC-9 and HSC-6 cells were treated with 50 mg/ml CHX, and RRM2 expression was detected via western blotting. (**I**) Western blotting analysis of the effect of lncOCMRL1 silencing on EMT in OSCC cells. (**J**) Schematic diagram of the molecular mechanism by which lncOCMRL1 promotes OSCC cell migration, invasion and proliferation by antagonizing RRM2 ubiquitination. (**K**-**L**) Representative images (**K**) and statistical analysis (**L**) of the correlation between lncOCMRL1 and RRM2 expression levels by ISH (scale bar, 100 μm). The error bars represent the SDs of independent experiments. * *p* < 0.05; ** *p* < 0.01; *** *p* < 0.001

After clarifying the biological function of lncOCMRL1 in regulating OSCC metastasis and proliferation, we further explored the molecular mechanisms regulating OSCC progression. LncRNAs with different subcellular localizations work in different ways. Most transcribed lncRNAs are transported into the cytoplasm, interact with proteins to exert their regulatory functions and are regulated at the post-translational regulation, thereby exerting protumor or antitumor effects [17–20]. We found that lncOCMRL1 specifically binds to a 40–50 kDa protein through RNA pull-down (Fig. 4A). Further mass spectrometry analysis revealed that this protein is ribonucleoside-diphosphate reductase subunit M2 (RRM2). The results were verified via RNA pull-down (Fig. 4B) and RNA immunoprecipitation (RIP) assays (Fig. 4C) to clarify the mutual interaction between lncOCMRL1 and RRM2. To explore how lncOCMRL1 regulates RRM2, we analyzed changes in RRM2 at different levels after lncOCMRL1 was silenced. We found that RRM2 expression was significantly reduced at the protein level (Fig. 4D) but was not altered at the mRNA level after lncOCMRL1 was knocked down (Fig. 4E), indicating that lncOCMRL1 regulates RRM2 via post-translational regulation. The ubiquitin proteasome pathway is a classic pathway that affects protein stability. It has been previously shown that RRM2 can be degraded through the ubiquitin proteasome pathway [21]. Therefore, we speculate that lncOCMRL1 regulates protein stability by binding to RRM2 and affecting its ubiquitination [22, 23]. The results revealed that the ubiquitination of RRM2 was increased after lncOCMRL1 was silenced (Fig. 4F-G). After lncOCMRL1 was silenced, the stability of the RRM2 protein decreased (Fig. 4H). The results revealed that when lncOCMRL1 is highly expressed, it binds to RRM2 and inhibits its ubiquitination and degradation.

### Downregulating lncOCMRL1 expression inhibits EMT in OSCC cells

We found that RRM2 regulates OSCC cell function. After RRM2 was silenced (Figure S4A-B), OSCC cell migration, invasion, and proliferation were inhibited (Figure S4C-H). RRM2 promotes EMT and promotes the invasive phenotype of tumor cells by activating the JAK2-STAT3 pathway [24–26]. After RRM2 was silenced, the EMT phenotype was suppressed (Figure S4I). We speculate that lncOCMRL1 affects EMT to promote OSCC cell migration and invasion. The results showed that after lncOCMRL1 was knocked down, the expression of E-cadherin increased, and the expression of N-cadherin, vimentin and MMP9 decreased (Fig. 4I), which was consistent with the direction of RRM2 regulation. The results revealed that when the expression of lncOCMRL1 was downregulated, EMT was inhibited, resulting in OSCC invasion and migration (Fig. 4J).

We also evaluated the correlation between the expression levels of lncOCMRL1 and RRM2 by ISH in tumor tissue samples. The results revealed that the expression level of lncOCMRL1 was positively correlated with the expression level of RRM2 (Fig. 4K-L). In summary, lncOCMRL1 specifically binds to RRM2 to maintain its stability, regulates RRM2 expression at the posttranscriptional level, accelerates EMT and promotes OSCC invasion and metastasis.

### NPs-mediated lncOCMRL1 silencing inhibits the proliferation, invasion and migration of OSCC cells

**Fig. 5:**
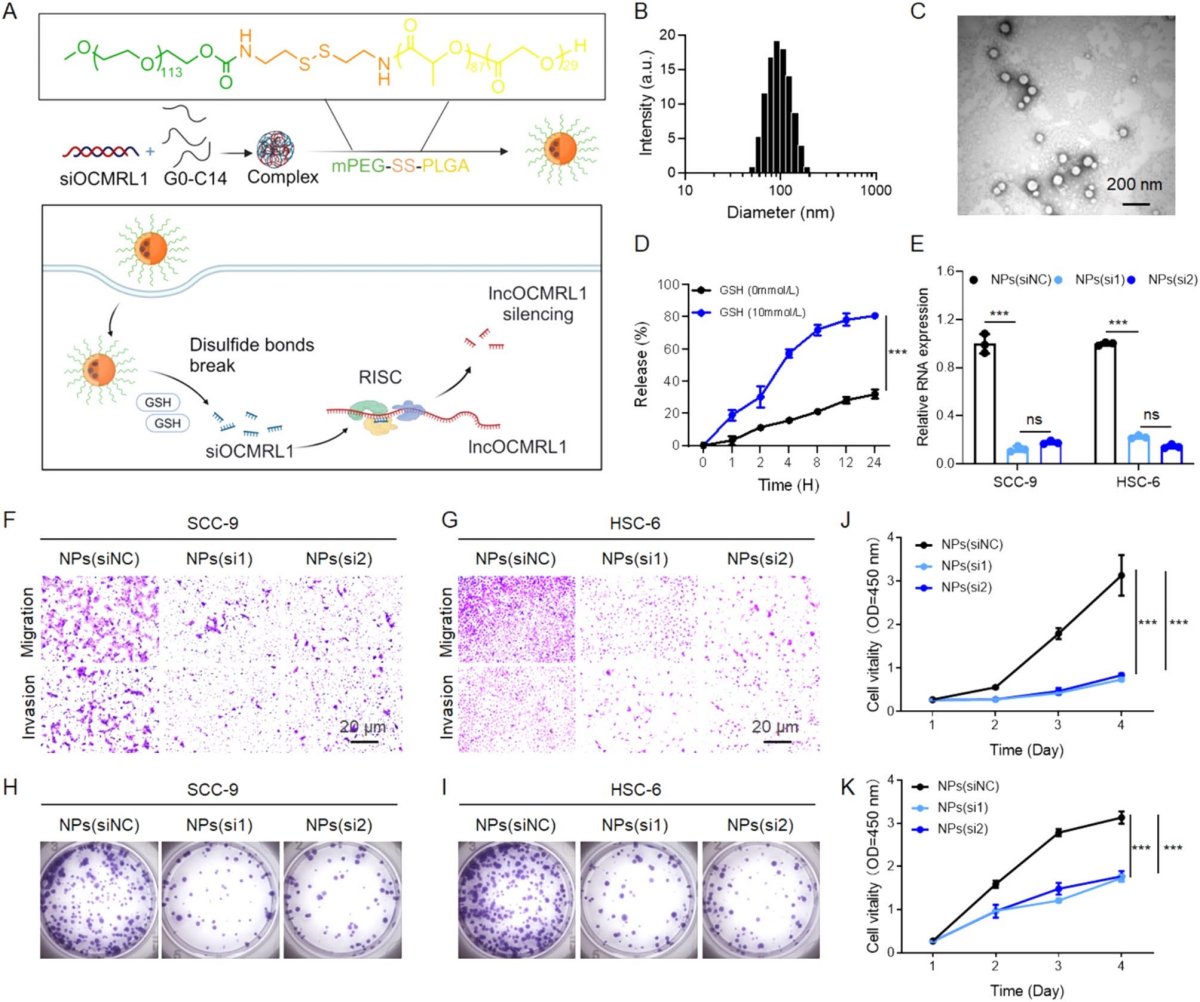
NPs-mediated lncOCMRL1 silencing inhibits the invasion, migration and proliferation of OSCC cells. (**A**) Schematic diagram of lncOCMRL1 silencing mediated by reduction-responsive nanoparticles made with the cationic lipid compounds G0-C14 and the amphiphilic copolymer mPEG-S-S-PLGA. (**B**) Size distribution of the NPs(siOCMRL1) in aqueous solution. (**C**) Electron micrograph of the NPs(siOCMRL1) morphology (scale bar, 200 μm). (**D**) Detection of siOCMRL1 release from the NPs(siOCMRL1) in the presence of glutathione. (**E**) qRT-PCR analysis of lncOCMRL1 knockdown in SCC-9 and HSC-6 cells using 50 nM NPs(siOCMRL1). (**F**-**G**) Representative images of migration and invasion after lncOCMRL1 was silenced in SCC-9 and HSC-6 cells via NPs(siOCMRL1) (scale bar, 20 μm). (**H**-**I**) Representative images of colony formation after lncOCMRL1 was silenced with NPs(siOCMRL1) in SCC-9 and HSC-6 cells. (**J**-**K**) Proliferation of SCC-9 (**H**) and HSC-6 cells (**I**) after lncOCMRL1 was knocked down via NPs(siOCMRL1). The error bars represent the SDs of independent experiments. * *p* < 0.05; ** *p* < 0.01; *** *p* < 0.001

Owing to the significant role of lncOCMRL1 in regulating OSCC metastasis and proliferation, we modulated lncOCMRL1 expression for tumor treatment. The regulation of lncRNA expression is challenging, and we used siRNAs to regulate the expression of this lncRNA at the RNA level [27, 28]. However, as a negatively charged biomacromolecule, siOCMRL1 is easily metabolized and degraded by RNase in the blood circulation in the body and cannot exert its function. Owing to the hydrophilic effect of PEG, the hydrophobic effect of PLGA, and the disulfide bond, it can respond quickly to the reducing substance glutathione (GSH). For this reason, we developed a methoxy-polyethylene glycol-b-poly (lactic-co-glycolic acid) polymer and a reduction-responsive disulfide bond (expressed as mPEG-S-S-PLGA) as a nanocarrier, which can quickly form a PEG hydrophilic shell and an internal hydrophobic structure with PLGA as the main body so that it can be stably transported in the blood when the contents encapsulated and will not be cleared. The mPEG-S-S-PLGA was used as nanocarriers; the complex was formed by encapsulating the amphiphilic cationic lipid compound G0-C14 (Figure S5) and the anionic siOCMRL1 through electrostatic interactions. The formation process of the nanoparticles (NPs) is shown in the figure, and they can quickly respond to high concentrations of the reducing substance GSH in tumor cells, resulting in the cleavage of disulfide bonds and the release of internal siOCMRL1, thereby silencing lncOCMRL1 (Fig. 5A, Figure S5) [29, 30]. The particle size was 100 nm (Fig. 5B), and the nanoparticles were spherical (Fig. 5C). NPs(siOCMRL1) had excellent glutathione (GSH) response properties (Fig. 5D). Moreover, the treatment of OSCC cells with these nanoparticles effectively downregulated the expression of lncOCMRL1 to 12% (Fig. 5E). Treated OSCC cells exhibited poor invasion, migration and proliferation (Fig. 5F-K). These results showed that the NPs have excellent knockdown ability in vitro.

### NPs-mediated lncOCMRL1 silencing inhibits OSCC tumor growth and metastasis

**Fig. 6:**
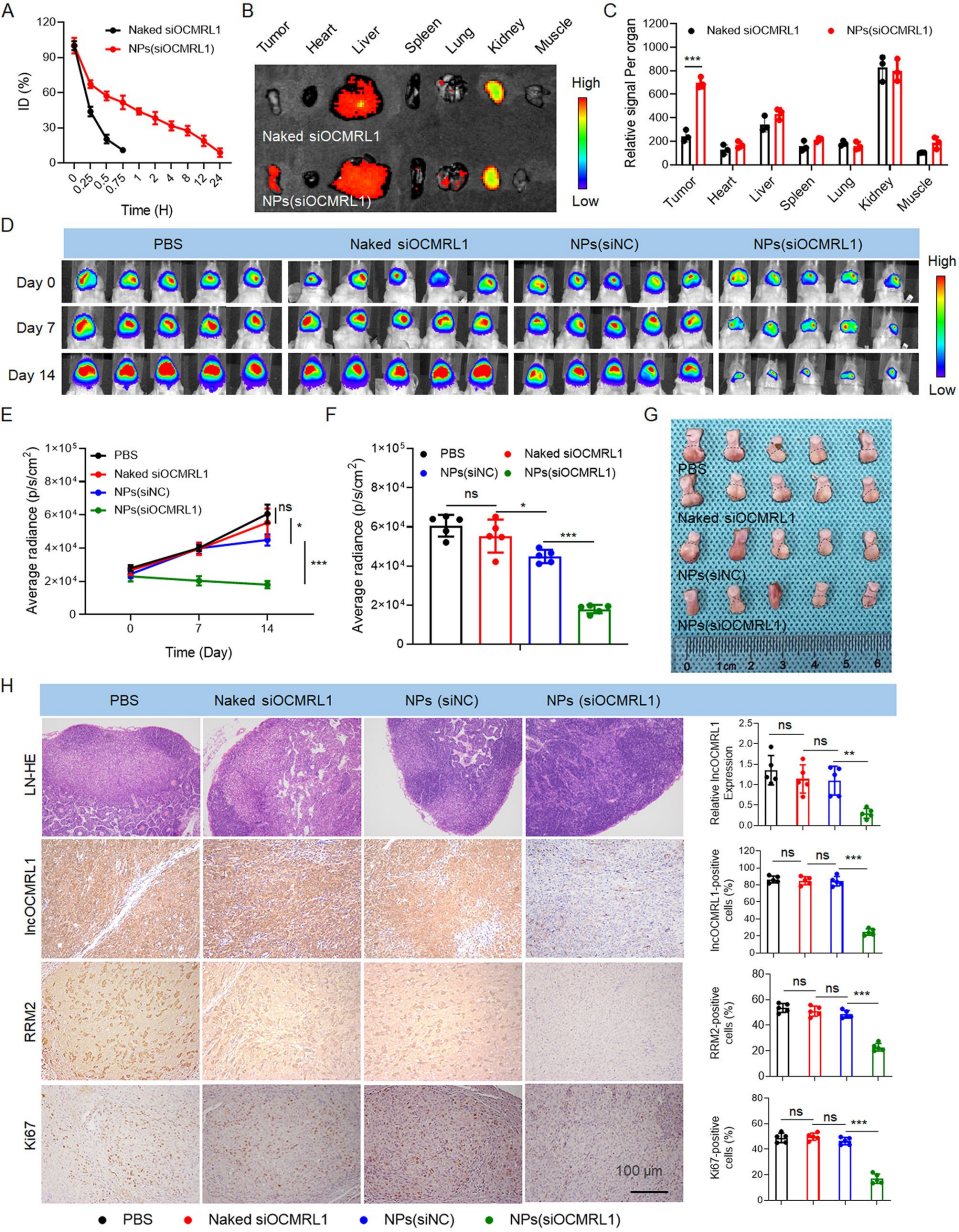
NPs-mediated lncOCMRL1 silencing inhibits OSCC metastasis and growth in an OSCC tongue orthotopic xenograft model. (**A**) Blood circulation time of naked siOCMRL1 and NPs(siOCMRL1) in mice. (**B**-**C**) In vivo imaging fluorescence images (**B**) and statistical analysis (**C**) of the enrichment of naked siOCMRL1 and NPs(siOCMRL1) in tumors and major organs in mice. (**D**-**F**) In vivo imaging fluorescence images (**D**) and statistical analysis (**E**-**F**) of tumor-bearing mice treated with PBS, naked siOCMRL1, NPs(siNC) or NPs(siOCMRL1). The mice were injected with nanoparticles via the tail vein for antitumor treatment three times every two days. (**G**) Tongue photo image of tumor-bearing mice treated with PBS, naked siOCMRL1, NPs(siNC) or NPs(siOCMRL1). (**H**) qRT-PCR results showing lncOCMRL1 expression in the tumor tissues of mice in each group after systemic treatment. Representative images of HE-stained cervical lymph nodes from mice. Representative images and statistical results of ISH and IHC staining for lncOCMRL1, RRM2, and Ki67 in tumor sections (scale bar, 100 μm). The error bars represent the SDs of independent experiments. * *p* < 0.05; ** *p* < 0.01; *** *p* < 0.001

After demonstrating that the NPs could effectively silence lncOCMRL1 in vitro, we analyzed the antitumor effects of the NPs in vivo. We analyzed the circulation time and tumor site targeting efficiency of the NPs(siOCMRL1) in mice. We found that after injecting the NPs(siOCMRL1) into the tail vein of the mice, the blood circulation time was prolonged, and 8% of the nanoparticles remained in the blood after 24 h (Fig. 6A). The intratumor enrichment of the NPs(siOCMRL1) was examined via tail vein injection into mice with orthotopic HSC-6-luci tongue tumors. In vivo imaging revealed that, compared with that of naked siOCMRL1, the tumor enrichment of the NPs(siOCMRL1) was greater (Fig. 6B-C). After confirming the long blood circulation time and high tumor enrichment ability of the NPs, we applied the NPs for antitumor treatment. HSC-6-luci cells were injected into the tongue of BALB/c-nu mice to establish an OSCC tongue orthotopic xenograft model. The tumors of the mice in the NPs(siOCMRL1) treatment group grew more slowly than the tumors of the mice in the control group did (Fig. 6D-G, Figure S6B). The body weights of the mice were measured every two days, and statistical analysis was performed (Figure S6A). Histological analysis of the tumor tissue revealed fewer metastases in the cervical lymph nodes in the NPs(siOCMRL1) group, indicating that the nanoparticles inhibited OSCC metastasis and growth in vivo (Fig. 6H). ISH and IHC were performed to assess the expression of lncOCMRL1, RRM2 and Ki67 in tumor tissues, and the expression of these factors was significantly decreased in the NPs(siOCMRL1) group (Fig. 6H).

**Fig. 7:**
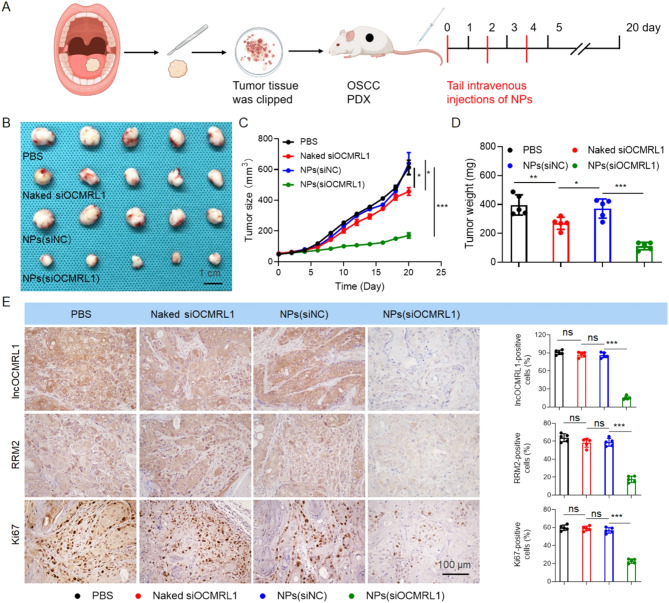
NPs-mediated lncOCMRL1 silencing inhibits OSCC growth in a PDX model. (**A**) Schematic diagram of the PDX model for tumor inoculation and treatment of PDX tumor-bearing mice. (**B**-**D**) Tumor images (**B**), tumor size change statistics (**C**) and tumor weight change statistics (**D**) for PDX tumor-bearing mice treated with PBS, naked siOCMRL1, NPs(siNC) or NPs(siOCMRL1). (**E**) ISH and IHC analyses and statistical analysis of lncOCMRL1, RRM2 and Ki67 expression in tumor tissues from PDX tumor-bearing mice in each group after systemic treatment (scale bar, 100 μm). The error bars represent the SDs of independent experiments. * *p* < 0.05; ** *p* < 0.01; *** *p* < 0.001

To further evaluate the therapeutic effect of the NPs(siOCMRL1) on clinical tumors, we collected fresh tumor tissues from OSCC patients during surgical resection to construct an OSCC patient-derived xenograft (PDX) model (Fig. 7A). We found that the NPs(siOCMRL1) significantly inhibited tumor growth (Fig. 7B-C). At the end of the treatment period, the tumor volume in the NPs (siOCMRL1) treatment group was reduced by nearly 60% compared with that in the untreated group (PBS) (Fig. 7C and Figure S7B), and the tumor weight was reduced by nearly 60% (Fig. 7D). The body weights of the mice were measured every two days, and statistical analysis was performed (Figure S7A). ISH and IHC confirmed that the therapeutic effect of the NPs was excellent in the PDX model mice, and the expression of lncOCMRL1, RRM2 and Ki67 was greatly reduced (Fig. 7E). Notably, no obvious histological lesions were observed in major organs after treatment with the NPs(siOCMRL1) (Figures S8A and S9A). In addition, peripheral blood was collected from the mice for hematological parameter detection. All the detected hematological parameters (ALT, ALB, ALP, UA, AST, and CREA) were within the normal ranges (Figures S8B and S9B). Overall, the experimental results showed that the NPs have excellent biosafety profiles.

## Discussion

Owing to the particularity of the growth site of OSCC, lymph node metastasis and local micrometastasis are prone to occur, and these processes lead to tumor recurrence and worsen patient prognosis. This is an urgent clinical problem that needs to be solved [31]. In most tumors, key genes have been used as tumor markers and therapeutic targets [14]. Therefore, there is an urgent need to explore targets that regulate OSCC metastasis and the expression of related genes to achieve good therapeutic effects.

The process of tumor metastasis involves genetic and cellular behavioral changes in tumor cells [32]. Epithelial-mesenchymal transition (EMT) is a classic process of tumor metastasis. The EMT program executes the metastasis cascade, and many signaling pathways regulate EMT, such as those involving transforming growth factor (TGF) and fibroblasts; these pathways include the cell growth factor (GF), Wnt/β-catenin and Notch pathways, but the specific upstream targets that regulate EMT are not yet clear [33–35]. RRM2 is a subunit of ribonucleotide reductase that catalyzes the formation of deoxyribonucleotides from ribonucleotides and plays an important role in cell cycle regulation and DNA damage [36, 37]. Previous studies have shown the important role of RRM2 in tumor cell chemoresistance [38]. In this study, we previously discovered the role of RRM2 in regulating tumor metastasis. We also innovatively discovered that lncOCMRL1 can bind to the proto-oncoprotein RRM2, which has been reported to promote EMT, maintain the stability of RRM2, and promote OSCC metastasis [24, 39].

LncRNAs have powerful biological functions, but because lncRNAs do not have the functional domain of a protein, antibody drugs and small molecule inhibitors cannot be developed to target them. Therefore, it is difficult to modulate the expression of lncRNAs [27, 28]. New tools, such as modified antisense oligonucleotides (ASOs) and CRISPR-Cas9 technology and its derivatives, are common ways to regulate lncRNAs, but their off-target effects and in vivo toxicity limit their application [9, 40]. To address this issue, we herein employed RNAi technology to regulate the expression of lncOCMRL1. As the effector molecule of RNAi technology, siRNA has been widely used to regulate the expression of target genes. However, siRNA is a negatively charged biomacromolecule that can be easily degraded by RNase and cannot cross cell membrane, and specific delivery vehicles are required to facilitate in vivo siRNA delivery [41]. In the past few decades, various types of nanoparticles have shown great promise for improvement of in vivo siRNA delivery, and several RNAi NP platforms have been marketed (Onpattro and Givlaari) or entered into early phase clinical trials [42].

In order to safely and effectively deliver siRNA systemically to tumor cells in vivo, we develop bioresponsive nanoparticles to have better anti-cancer effects [43–45]. Among them, because the concentration of GSH in tumor cells is extremely high, we developed reduction-responsive nanoparticles to inhibit tumor growth and metastasis. The nanoparticles have the following characteristics: (i) compared with naked siOCMRL1, the hydrophilic PEG shell of the NPs(siOCMRL1) can greatly prolong the in vivo circulation time of the siRNA. (ii) NPs accumulate in tumor tissues through the high permeability and long retention effect (ERP effect). (iii) responding to the high concentration of GSH in tumor cells, leading to the rapid release of siOCMRL1, silencing siOCMRL1 and blocking the RRM2/EMT signaling pathway, thereby achieving the effect of inhibiting tumor growth and metastasis.

Our results showed that lncOCMRL1 is highly expressed in highly metastatic OSCC cells and OSCC tumor tissues and promotes the growth and metastasis of OSCC. In terms of the molecular mechanism, lncOCMRL1 binds the proto-oncoprotein RRM2 to maintain its stability and promotes EMT in tumor cells, thereby inducing tumor metastasis and growth. Therefore, we applied a reduction-responsive nanodelivery system to deliver siOCMRL1 to provide an efficient treatment for OSCC metastasis and growth.

## Conclusions

In summary, we discovered a key regulator lncOCMRL1 in OSCC metastatic tumor tissues and cell lines. LncOCMRL1 downregulation inhibits OSCC growth and metastasis by promoting RRM2 ubiquitination and inhibiting EMT. To increase the translational potential, we developed a reduction-responsive nanodelivery system to deliver siRNA and found that the nanoparticles had high antitumor ability in an OSCC tongue orthotopic xenograft model and a PDX model.

## Electronic supplementary material

Below is the link to the electronic supplementary material.

Supplementary Material 1

Supplementary Material 2

## Data Availability

The datasets used for the current study are available from the corresponding author upon reasonable request. All the data generated or analyzed during this study are included in this published article or its supplementary information files (Figure S1 - S9) and Supporting Information 1.

## Abbreviations

lncRNAs: Long Noncoding RNAs
OSCC: Oral Squamous Cell Carcinoma
lncOCMRL1: Oral Squamous Cell Carcinoma Metastasis-Related lncRNA 1
TCGA: The Cancer Genome Atlas
OS: Overall Survival
DFS: Disease-Free Survival
RRM2: Ribonucleoside-Diphosphate Reductase Subunit M2
EMT: Epithelial to Mesenchymal Transition
NPs: Nanoparticles
GSH: Glutathione
PDX: Patient-Derived Xenograft
