# Supplementary material for: LncOCMRL1 promotes oral squamous cell carcinoma growth and metastasis via the RRM2/EMT pathway
Source: J Exp Clin Cancer Res. 2024 Sep 30;43:267. doi: 10.1186/s13046-024-03190-w (PMC11441159; doi:10.1186/s13046-024-03190-w)
Supplement: Supplementary file 1 — Supplementary Material 1 [file 13046_2024_3190_MOESM1_ESM.docx]

lncOCMRL1 sequences

GAGCGGGGAGCAAGGCCTGCGGGGAGGCGCAGGATGGACGCGTTGGCTGTCATGATGTAGGACCTCCTGACCTAGAACCATGCCCGGCGCAGGGAGAACAACGAGCTTACGGACCGGGTGCGGGGGCTGCTGTGCGAGAAAGCCTACCTGCTGGCCCGGGAGCGTTCGCCGGCCCACCTGGTGGCTTTCCCTGGACGTTTAACGGCGACTCCGCCCGGCCTCCCGACTTTTTGATGCAGGCGTTCTCTTACATGACTTTCTTCGAGGCTAGATTCTCGAAAGACATCCTGAAGGTGGCTTTCTTAATTAGCTGCCTCACCGGCTGGCGGAGCAGTTCGTAGTCGCCTACATCGAGAGGGAGAGCCCCGTCCTGGCTCAGTACTGGGGCTTCGTGGACGCGCTGTAGCGGGTCTTCGCCGTCGTGGGTAGGAAACAGTCGGGCCGGATCCCCGGTGTCTGGGGAGCGGCTGCCGGCCGGGCCCGCGCGAGCCGCTTCACTCCTACAAGCCCGGCTCACTAGTTCCAAGCTGCTGGACTTTGACCCTCGCTGGGAAGCTTGATCGCCGCCTGTTCTCGCCAATCTCTATGCCTTTGCACTGCCCAGCAACCCGGTCACTGCCCAGGACCTGTTAAAAGTTGAATGGACCTCCCAGAATCACGCCGCTGCCACCGCCGTTTCTAGAGACGGCTTTTGGCCACCTTTGAGAATCAGGGTCAAACTTGGAAGGGCCTGCATTTGTCACACTTCCTCGGACACTAATTTGGATAGTTCAGGCCCAGAGACGGCTTCCCGGGTTAGTGTGCCTGACAACACTGAAGAAAAATTGTGACATTTTCCTTCCCCAGAATTACTTCGTTTTATACATTTCACTTCTCTTCTGAAATCACAGCAATGCCAGTTTGCGCCTTTTGGTCTGAGGAACGCAGGCCCTGGCCACTACCAGGAAAAAGTATTTACTTTTAAAACTTGAATGTATATCTTTACCGTATGCCTAGTACAGTAGTGCTTGGAAGAAACAGATTAGTAAGACAAAATATATGTCTTCAAGAACACTTACGTTCTGAGAAAGAATTCTGAATGCAGATTCTAATGTCTGGTAAAATGCTTTACAGTGTAAAGAATGCATATATGATTCTTATTTTATGGCATTTTAAACTTTCCACCCCACGGTTTTAAAGTGTTAATATATGTTAAAAATATATGCAATTTATAAAGGAGAAGGAAAGCCCAGCTTGATTTTTGCTGCCAAAGATGGTATATTTAAAATTAAACGTAACTAATGGCAAAAAATAATGGGAATGGTGCAGTCTTTGAGCTGTATCCAGTTGGCATTATTCTCTTGGACTAAAAGGAATAGTATTAACTTCCTCACTTTGGGTAAGCCACTTTAGATTTTTGGGCTGCAGTTTTCTCAGGTAATTAGAGACTTTATATTATGAAAGTTTGGTTCACAATTCTTTGAGTCTCTTTTGGGAACAAAGAAGGTAAGAGGAGGATATGTGCCCTCCTCTCAATCAAGGGGTTTCTTGAAACCCTCTAAGGAGAAAAATCTGAAACTGCCTTTCCTAAAACGTTCATGGGTTTGTCATTCTGATGATCAATATCGTCCCACTGAAGTATCTTTTTGAATTCCTAGTTAGGAATCTTTATGTTAAGATAAACTGATCACTCTCTCCTTCGTGGACAGTTTATCTACCTAAAGATGATAGGGGAAAACACTGTTTTTGAAAGACGAAATAAAGACATGTTGTAGCAGTTATTTTATTGCCAGTTAGTATTACAAAAGTTCCTAAGGAACTCCCCATTAACTTGGCCTCATATGAACTACGCTTGAGTCCCGTTTTAAATTGAGTCTTGATCGTATTTACTGACAGTTTCAAAGCAGCGTTTTTTTTTTTCCTCCATAGTAAAAAACCTACAGGATACCACCACCAAAGGAAAAAACAAGTTTTTTTCAGTAAACCTCACAAGAGCATACATTTGATAGAATACACGGATTTTCTAATGAAAGCTCGCCGTCACCATTTTATAAGTCAGAACATGAAAGTCAAAATATCTCATACAGATGTCTTTTTTTTTTGAGAAGGTACACAAGGCTTGCTTGTAGAGATGGAGTTACATGGAGGGACAGAAGGTCACTGGTCAGTGATTTTAGGTTTTTATACTTCTGAAAACAGGTAATTACAGATTCTGATCAGTTTATCATTTTCTTTAGTCTTTTATTTTCTGCTGAATGCTCTCAAGGGAACGGATCCATACCTGTTTATCAGGCTCACCTGGAAGAGCCAAGTCATCCACTCAGGTCTTCCTCTGGACCTGCATAATTTCCTCATTTACTAGATTGGGTTTCTTTTATGTGTGGGCTTTTAATGCTTGGTATCTCTGGTCTTCCTAATATGTAATGCTTAGCATAATAATTTTAGCCATTATTCATTTTTGTAGTAGGCTTCACTAGCTTCCTGCTTCTCAAATCCCTTGAAAAGATGAGATTTTTTTTTCGTGGAGTTCACGTCTAGTAAGTTGGTCTTGATTTTTTTTTTCTTTCAGAACCTTCTGGATTTCAGCTTTGGCAAGCTTCCTCTGTTTCTGAGACACCTCCATCACCTGGGTTACAATCCCATCCATGATTTTCCCCATCTCCAGGCATTTTTCATGGAAAGCTCCTCATGCTTTTTAATTCCAGTTTATCCATTTGTGTGGCTTTAACCTCCACATCCAGGTCTTTCTGGCTAAACTGCAGTATATCCACAATGGGGCCAATGGACACATAAGGGAGGCCCTATCACAGGAACACGAACACCCGGAGTCGCTAGAAATCAGATTCCACCGGCTTTGCTTCAGGCCGACTTCCTAAGGAAGGCATGGTAGCATCACAGATGCTGTTTGTCTCAGTTTCCTGAGAAGCCAGTTTCTTAGAGCTGTCATTCAAGAGTGGGTCAAATTTGAGATATAAAAACCTTTCTCAAGGTAGACTCTTTGGCCCCTATGCCTAGTATGTCAGCTGGGCCTCTGAAGCTCTCCTCTTTGAGGTCCTGGCAGGCTCTGGCCCCTGTTGAGAAGGGGAAGCAGTCATGTGAACTCATGTGCTGGAAGAATCCACTCTACCCCTTCCCTGGCTTCTCTGCTGCAATTTTTGTCTTTAAACGTATTTTCAGGTGATTCCAGTTTGCATACATAATTGAGAAATATAAGATTTGGTCCCCAGTAGAGAGCCAGGTGAACTTAAGAAGGAGTCCATCTAAGAAAATTGCACAATTATCTAGTTTCACTTGGGTTTAGCTTTTATGGCTTACTTGATTTAGTAATCAAATGATTTTCAAATGATTTTGATCAGGTGACTCTTCGTAAACTAGAGAATGAACTTGAGAGCCGAATTAGCACAGTTTAAAAATAATCATTTAGTTTTTGTTCTCTATTGTGTAAATTATTGAGGTTTGACTGTCTTGGCTAAACTAAGGCTCATCTAATATCTTTATTTTTGATATCCTAATTTCTTGCCAATTAATTGAAGGTAACATACTAGCAGACCAGATAACTACTTATGCAAACCTCAGATAACCACTAATTTAAACAAAATTAAATCCTTTGCCTTCCCCAGATCCTTTGCACATGGCCTGCTATCAACAATCCTCTACAATGATTCTCAGCATCACCTAGGGAGCTTATGTAAAATCTCAAATTCCTGGGCCCTATCCCCAGAGTTTCTTATCAAGTAAGTCTGCATTGGGATCTGAGGATTTGCATCTCTAACAAAAATTCCTAGGCGATGCTCACACTGTTGGTGTAAGGGTGAGTACAAATTAGAAATAAAACTATTTAATTCTGCCTGGTGCAAAGGAGAGAGAGAGACTTTTACCCTCCACATCCTTTTCTTTGGTCATTTACTTTAGGAAACTTAATTGTAAGCTTTTTCTGTGCCTCTTTGAAATGCCTGTAAATCTTTCCAAAAACTAAATTAGGCTTTTGCCAATTTTACAACCTAAGAATGTGAATTCCTCAAGGACCTGGGAGCTATCTCACTGAAATTTAATCATTGAGAAAAATAGTACCCCTATCTTTCAGCTTCCTGGAAACATAAGAGCCTAACTTTATTAGGCACCTTGCTCCATTACAAAGCTATCTTCTGTCATAAAGATACAAGAAATTTATTTTTCCCTTGGACAAAAGCAACTAACTAGCAGAGGGTCACCAAAATTACCAGGTGAATTTAGGGTGAACTCTGTGTGACAAATGGTGCTGCCAAGTTCTCTTGAGTACTATAGAAAGTGCATGACATGAATTTTTTACTTCATGATGGTGCAAAACCATTGCTTTTCACTTTCAGTATAATATTCAAGAAATTACATATTGAATATAAGATATTCAATATCTTATTCTTTATTATAGGCTTTGTGTTATATAATTTTGCCCAACTAACTGTAGGCTAATGTAAGTGTTCTGAACATGTTGAAGGTAGGTTAGACTAAGCTCTAATGTTCAGTAGGGTAGGTGTATTAAGTGCATTTTCAACTTACGAAATTTAAGCTGATATCCAAAGAATGAGTAAGAATGAGGTAAAGGAGCAAGAAAACATTGCTTCAGGCAAAGGAATAGTATGTGCAAAAGCCCTGGGGTTTAAGAATATGATGTTTTTAGAGAACTGCAAATAATTCAGTATGGTTGGAGCAGAGGGATGTGGAATGATTATTGATTCTGTTGGAGAGTAGACAGAGGCCAGATTGCTATGCTTAGATACAATTTCAACCACCTACAAATCCATCTAATTACCTACATGACCATCATCATCTGGTCATGTTTCTCCATTTTATCATTATGTATACCTTAAAGACTTGCTCACATTACTTAAAATTGAGATAGGTTAGGCATCTATCCAATCTCCCATGCCAGTAATTCTACAGAAACAGATTAGGTTAGCTAGATAAGCATGTTTACAATTAAATTTATGCTGTATCCTTCTAATCACCACTTTCTCATCAAAATGCTCACAAATCATCTGCATCATTGTCCATTCTTTAAAAATGTATAAACCCATCTCTACTAAAAATACAAAATTAGCTGGGCATGATGGCGCACGCCTGTAATCCCAGCTGTTCAGGAGGCTGAAGCAGAAGAATTGCTTGAACCCCGGAGACGGAGGTTGCAGTCAGCCGAGATTGCACCATTGCTCTCTAGCCTGGGCAACAAGAGCAAAACTCCATCTCAAAAAAAAAAAAAAAAGTATAAATTTGTATCAAGCATGCTGGCTTGAGGGTTTAAGGAATTTCTTTTTTTTATTTCTGACATACTTCTGTGGAGTGTGTTTTGCTTTCATTTTTGTTGTATTGTTTGTAATTTACACTATTAACAGTTTCTATTAGTGCTGTGGAATTAATTCCTCTTGTGCTGAAGATAGAAATATATTATTTCTAGGAAGAAATTGTCCTTATCGTCTTCACTAATATTGGACTTAGAGAGTCCTCCTATAAGACATGTTCTGTTTTTCATTTTGAACAGTTTCCTTGATGGAGAACTATCAAAGCAGTCAAAGCTATCAAAATAAAAAAGAATTCTGATTGGGTGCAGTGGCTCACACCTGTCATTCCAGCACTTTGAGAGGCCAAGGTGGGCAGATCACATGAGGCCAGGAGTTCAAGACCAGCCTGGCCAACATGGCAGAAACCCCATCTCTACTAAAAATACCAAAATTAGCCGGGCATGGTGGAACACACCTGCAATCCCAGCTACTCGGGAGGCTGAGGCAAGAGAATCGCTTGAACCTGGGAGGCAGAAGTTGCAGTGAAACAAGTTGGTGCCAATGCATTCCAGCCTGGATGATGGAGCGAGACTCTGTCTCAAGAAAAAAAAAACAAAAACAAAAACAGAACTCTGCCTCATAGCGTCTTTTAAGTTATACACTGGACCTATCCTTTCCTCACTGACAGACATTTTAAAAATTTTTTGGTAAGGCCTAGTTCATATAAAATGTAATCCAAGCCAAAAGTTAACAAGAATAAGGGGAGGAAAGGGGACTCCAATAGCAGAGAAAGGTATTTACCTGGGATATACACTGCAAGAAAATCAAAGCTATAAGAAACGTCCATGAATAGTAGCCATAAGGCATCAGAGTGATAAAATTCCTGTCCCTAGGAGGGAATATTGGAGTTTGCCAGAGAAACAGAATGAGAGAGACAGAGAGGTTTATTGTAGGAATTGGCTCATATGATTACGGAGGCTGAGAAGACCCACGATCTGCCATCTGCAAGCTGGAGAATCAGGAAAGCTGGAGGTGTAATTCAGTCAAGTCCAATGGCCAGAGAAGCAAGTGTACTGATATCCAAGAGCAGGAGAAAATAGATGTCCCAGAACAAGCAGAGAGGCTGATTTTGTCCTTCCTCTGCCTTTTTGTTTCATATGGGGCACTGAATGGACTGATGCCCATCCACATTAGTGAGGGTGGATCTTCTTTACTCAGTCTACCAGTAGAAATGTCAATGACTTCCAGAAACACCCTCACCAACACACGTGGAAATAATGTTTTACCAGGTATCTGGGCATCCCTTGGTTCACTCAAGTTGACACAAAATTAACCATCACAGAAGGAGACTGGCCTTACTCTGAAATTAGGAAACTAAAGAAGTGACCAGAATGGAGACTAGGTAGAGACAACTAGTTCTCTACCAAACATGTACAGTTATTCGTTGGTATCTGAAGGGGATTGGTTCCAGGAACTCTCAGGGATACCAAAATCTGCAGGTGCTCAAGTCATTTATATAAAATATTACAGTATTTGCATATAACCTTTGCACATCTTCCATATACTTTAAATCATCTCTACATTACTTATAATAATGAATGTGTAAATGCTATGAAAATAGTTACTACACTATTGTTTATTTGTATTTTTATTGAATTGTTTTGGGGTGGGGGGCAGCTGTATCTTTCTTAGTAATAGAACCCCTGGTTTTAGCTGGGCACATGACTGCCCTCAATAAAGATTAAAGTACCCCAGCCTTCCTTGAGATTGTGGCCATGTGACTGAACTTTAGACAGTGAGATATAAGCAGATATCTTCTGTGGCAGTGTTAGGAAACTATTAAAGACAGTAAGAACATTGCCCTTTGCCTTCTTTTTCCTTTCTTCATTTTTCTGCCTGGAATGCAAATGTGATGGCTAACACCCTAGCAGCCATTTTAGATTATGAAGATGAGAGTCACTCCCCAGGAAGGGTGGACCTGAAAGCTGGATTCTAGATGTGGAATGACAATACCAGCCCTGGACTACCTACCTGTAGGCTGTGTGTGTGTGTGTGTGATTGACAGAGAGAGAGAGAGATAGATACCACAAGTAACACAAAGCATCATACCTGGGACTGGGAAGGTTAATGTTTAAACATTTTAAATGTGGCATTGGTTTGTTGAAGACAGTCAAGCAGTAGGTGCTGCAGGCTTTGTTGAGGCTAGAAAGCTGTTGGTCTTTATTAGGTGATGGCAAAACAATGGATCACATTTTCATGTGCTGTGTCTTGGGAGGCAAATGATGAGAATGTAGGATTGGGGAAAATGGTAAGGAAAATCCAAAATGTTCATGTTTTCTAACCCCTTCTTGCCAATCTTAAAAGAAGGACAAACAGGCTAGATTAAGCAAGTCTACAGTTACAATTGGAAGGAAACACAGCCGTACTAAGGAAGTACTCTGTCTGTGGCCTGCAGTCTAGTTTGACTAGGTCCTGTATTTGAGCTTTGAAAGAGTAAAGAATACAAGTGCTCGTATACCCCTGAAGTAGTTACCAGCAGTGACTGCAAGGAGGTAATCTTATGGGATGAAACTATAACTCTGTATTAACCATCATATATAGCATATATGTGTATATGTATGTGTGTGTGTGTATATGTGTATATAAATATATGTGTGTGTATATACACACATGTACACACCCTACAACCCAGTGATTTCAAAAAATTAAACATTTGTTTAGCTCATGAATCTGTGGGTTGGGTGAACATTTCTATTGGTCTTGGCTGGGCTCACCTGTACCCAGTCAGCTGCAGGGTCTAGCTCTTCTTATCTTGGCTGGGATCTCACACATGTCTAGGATCAGCTGCTTTACTAGTCTAGGATTGTCTTGGCTGAGACAGGTGGACTACTCAGTTCTCCTCCACTGCCTTATCCTTCAGCTGGCCAGCCCAGAAATGTTGCCATGTTTACAGCATAAGAGCAAGCAAGAAAGAATAATATGCAACCACTTTTCCAAGCCTAAGGTGAAAGGGCGGGCAGAACCTGTGGTGTTAGGAGGCAACTCATACCAGCCCATGGGTCTACCGTTTAATTTTCAGAAATTTGGCAAGCTGATTGTTAACACAGCTGTTGTTTAATTTATGTACTTGTAATTAAATAATTTACATTAAAACAAAGATAATAGGTACTCAAAA
