# Supplementary material for: LncOCMRL1 promotes oral squamous cell carcinoma growth and metastasis via the RRM2/EMT pathway
Source: J Exp Clin Cancer Res. 2024 Sep 30;43:267. doi: 10.1186/s13046-024-03190-w (PMC11441159; doi:10.1186/s13046-024-03190-w)
Supplement: Supplementary file 2 — Supplementary Material 2 [file 13046_2024_3190_MOESM2_ESM.docx]

**LncOCMRL1 promotes oral squamous cell carcinoma growth and metastasis via the RRM2/EMT pathway**

**Supplemental Methods & Figures & Table**

**Methods**

**1. RNA extraction and quantitative real-time PCR (qRT-PCR)**

Total RNA was extracted with TRIzol, and after removing genomic DNA, 1 μg of RNA was reverse transcribed into cDNA. A SYBR Premix Ex Taq II kit was used for qRT-PCR analysis. The reaction conditions were as follows: 94°C for 2 min; 40 cycles of 94°C for 20 s, 58°C for 20 s, and 72°C for 20 s. All qRT-PCR experiments were performed on a Roche Light Cycle 480 II instrument. The primer sequences for PCR were as follows.

| Name | Forward | Reverse |
| --- | --- | --- |
| lncOCMRL1 | TGTGCGAGAAAGCCTACCTG | GCCTGCATCAAAAAGTCGGG |
| RRM2 | GTGGAGCGATTTAGCCAAGAA | CACAAGGCATCGTTTCAATGG |
| GAPDH | GAGTCAACGGATTTGGTCGT | GACAAGCTTCCCGTTCTCAG |
| MALAT1 | GCTTGAGAAGATGAGGGTGTTT | GTAGTGAGGAATAGGGCTTCCA |
| PVT1 | CCTGGTGAAGCATCTGATGCACG | GCCAGGCTTTGTGGCACACGC |
| LINC00662 | TTTGCCTTGTTCCTGAGCTT | CACCTCATGGATGCAGAGAA |

**2. In situ hybridization (ISH)**

ISH was used to detect the expression of lncOCMRL1 in tumor sections. Paraffinized tumor sections were heated to 60°C for 1 h, dewaxed with xylene, rehydrated with alcohol at different concentrations, and washed with tap water. Sections were treated with pepsin for 10 min at room temperature and incubated with 500 nmol/L of a biotin-labeled lncOCMRL1 probe (5′-GACTGTTTCCTACCCACGACGGCGAAGACC-3′) at 55°C for 4 h. After washing with phosphate-buffered saline (PBST) containing 0.1% Tween 20 and blocking with 10% FBS for 30 min, the sections were incubated with an anti-biotin secondary antibody overnight at 4°C. Subsequently, the slides were washed with PBST and incubated with IgG-HRP antibodies for 1 h. After adding horseradish peroxidase (DAB) chromogenic substrate and hematoxylin, the stained sections were finally observed under an Olympus optical microscope.

**3. Rapid amplification of cDNA ends**

Total RNA was extracted from the cells using TRIzol, and 5’ RACE and 3’ RACE were performed according to the instructions of the SMARTer RACE Kit (Clontech Laboratories, USA). The RACE PCR products were separated on a 1% agarose gel and further sequenced in both directions. The following gene-specific primers (GSP) were used for PCR: 5′-CGACTGTTTCCTACCCACGACGGCG-3′ (5′ RACE GSP1), 5′-CTCGATGTAGGCGACTACGAACTGC-3′ (5′ RACE GSP2), 5′-GTTCATGTTTTCTAACCCCTTCTTGC-3′ (3′ RACE GSP1), and 5′-CTCTGTCTGTGGCCTGCAGTCTAGT-3′ (3′ RACE GSP2).

**4. Isolation of nuclear and cytoplasmic RNA**

Isolation of nuclei and cytoplasm was performed using a Thermo Fisher kit (PARIS™ Kit, AM1921). After obtaining RNA, reverse transcription and qRT-PCR were performed as described above. GAPDH was used as the internal reference for the cytoplasm, and MALAT1 was used as the internal reference for the nucleus.

**5. Immunofluorescence in situ hybridization (FISH)**

Cells were seeded in confocal dishes (40,000 cells/well) and cultured for 24 h. After fixation with 4% paraformaldehyde for 20 minutes, PBS containing 0.1% Triton X-100 was used to permeabilize the cells for 10 minutes. Precooled gradient ethanol (75% ethanol at 4°C, 85% absolute ethanol at -20°C) was used for dehydration. After prehybridization with prehybridization solution for 1 h at 37°C, a 20 nM digoxigenin-labeled oligonucleotide probe was used overnight hybridization. The samples were washed with 2x SSC for 5 min at 42°C, which was repeated 2 times; the samples were washed with 2x SSC containing 50% formamide for 25 min, which was repeated 3 times; the samples were then blocked with 0.5% BSA for 1 h and incubated with an anti-digoxigenin-FITC antibody at 4°C overnight. Finally, after the cell nuclei were stained with DAPI, they were observed using a Zeiss confocal laser scanning microscope (CLSM). The sequence of the oligonucleotide probe for lncOCMRL1 was 5′-GACTGTTTCCTACCCACGACGGCGAAGACC-3′.

**6. Western blotting**

Cells were lysed with RIPA strong lysis buffer (containing protease inhibitors and phosphatase inhibitors). The protein concentration was determined using a BCA kit, and the proteins were denatured at 95°C for 10 min. After 10% SDS-PAGE, the proteins were transferred to a 0.45 μm PVDF membrane and blocked in TBST containing 5% skim milk at room temperature for 1 h, after which the membrane was incubated overnight at 4°C. The primary antibodies used were specific for RRM2 (Proteintech, 11661-1-AP-50UL, 1:1000), E-cadherin (Proteintech, 20874-1-AP, 1:5000), N-cadherin (Proteintech, 22018-1- AP, 1:2000), MMP9 (Proteintech, 10375-2-AP, 1:1000), vimentin (Proteintech, 10366-1-AP, 1:2000), GAPDH (Proteintech, 60004-1-Ig, 1:1000 ), and ubiquitin (Proteintech, 10201-2-AP, 1:1000). After the membrane was washed three times with TBST, the membrane was incubated with the secondary antibody for 1 h at room temperature. Finally, enhanced chemiluminescence (ECL) solution was used for luminescence and color development.

**7. Ubiquitination assay**

SCC-9 and HSC-6 cells were transfected with siRNA at a concentration of 50 nmol/L for 48 h. MG132 (10 μM) was added to the fresh culture medium, after which the cells were incubated for 8 h. The cells were subsequently digested with trypsin, after which the total protein was extracted using NP40 lysis buffer. The lysates were bound to Protein A/G magnetic beads with RRM2 antibodies overnight at 4°C, and the eluted proteins were detected via western blotting.

**8. Protein stability assay**

SCC-9 and HSC-6 cells were transfected with siRNA at a concentration of 50 nmol/L for 48 h, and then 50 mg/ml CHX was added at 0, 2, 4, 6, and 8 h to harvest protein samples and perform western blotting analysis.

**9. Glutathione-responsive release of NPs**

NPs(Cy5-siOCMRL1-1) were dispersed in 1 ml of PBS (pH 7.4). The NPs(Cy5-siOCMRL1-1) were subsequently transferred to a Float-a-lyzer G2 dialysis device (MWCO 100 kDa, Spectrum) immersed in PBS (pH 7.4, 0/20 nM GSH). At room temperature, 5 µL of the solution was removed at different time points and mixed with DMSO, after which the fluorescence intensity of Cy5-silncOCMRL1 was measured using a microplate reader.

**10. Pharmacokinetics**

Healthy BALB/c-nu mice were randomly divided into two groups (n = 3) and intravenously injected with (i) Naked Cy5-siOCMRL1-1 or (ii) NPs(Cy5-siOCMRL1-1). The siRNA dose for each mouse was 1 nmol. At predetermined intervals, orbital venous blood (20 μL) was drawn into a heparin-containing tube, and the wound was pressed for a few seconds to stop the bleeding. The fluorescence intensity of Cy5-silncOCMRL1 in the blood was determined via a microplate reader.

**11. Biodistribution**

After generating HSC-6-luci tongue orthotopic tumor mice, the mice were randomly divided into two groups (n = 3) for intravenous injection of (i) Naked Cy5-siOCMRL1-1 or (ii) NPs(Cy5-siOCMRL1-1). Twenty-four h after injection of a 1 nmol siRNA dose per mouse, the mice were imaged using an IVIS Lumina III (Perkin-Elmer, USA) imaging system. Organs and tumors were then harvested, imaged, and statistically analyzed.

**12. Immunohistochemistry (IHC)**

The dewaxing and rehydration procedures for the paraffin sections were the same as those for ISH. After the sections were repaired with EDTA antigen retrieval solution (pH 9.0) at high temperature for 40 minutes, the slides were blocked with 3% peroxidase blocking buffer for 10 minutes. The cells were incubated overnight at 4°C with diluted primary antibodies (Ki67: 27309-1-AP, 1:5000; RRM2: 11661-1-AP, 1:100). The slides were then washed and incubated with peroxidase-labeled polymer for 30 min at room temperature, washed and stained with DAB + substrate-chromogen solution and hematoxylin, and observed under an Olympus optical electron microscope.

**13. Biosafety testing**

Healthy BALB/c-nu mice were randomly divided into two groups (n = 3) and intravenously injected with (i) naked Cy5-siOCMRL1-1 or (ii) NPs(Cy5-siOCMRL1-1). The siRNA dose for each mouse was 1 nmol. Fourteen days after tail vein injection of the nanoparticles, the main organs of the mice were collected and subjected to HE staining. The venous blood of the mice was collected, and the serum was collected to detect physiological and biochemical indicators.

**Supplemental figures**


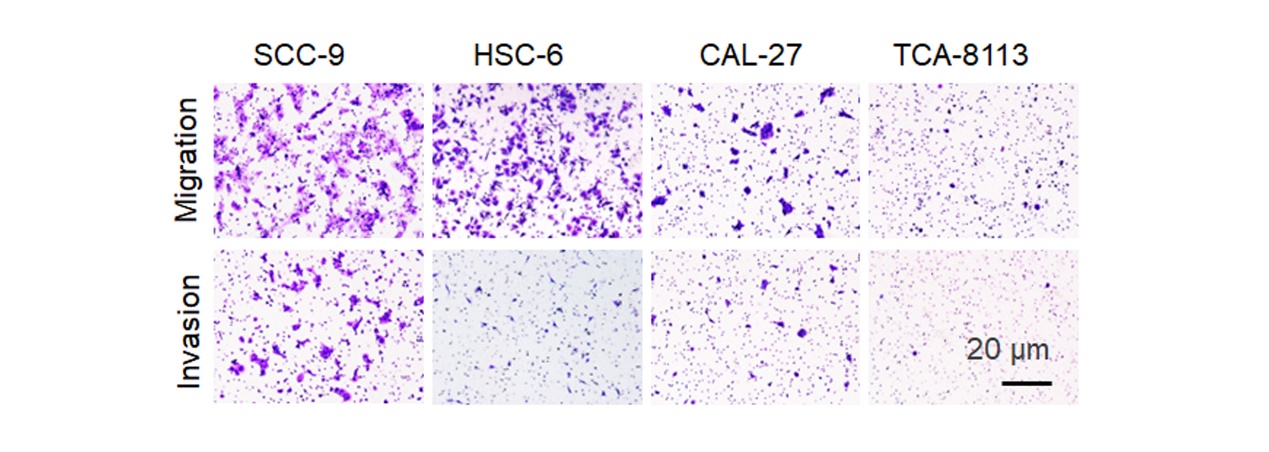


Figure S1

Representative images of transwell assays showing the migration and invasion abilities of SCC-9, HSC-6, CAL-27, and TCA-8113 cells (scale bar: 20 μm).


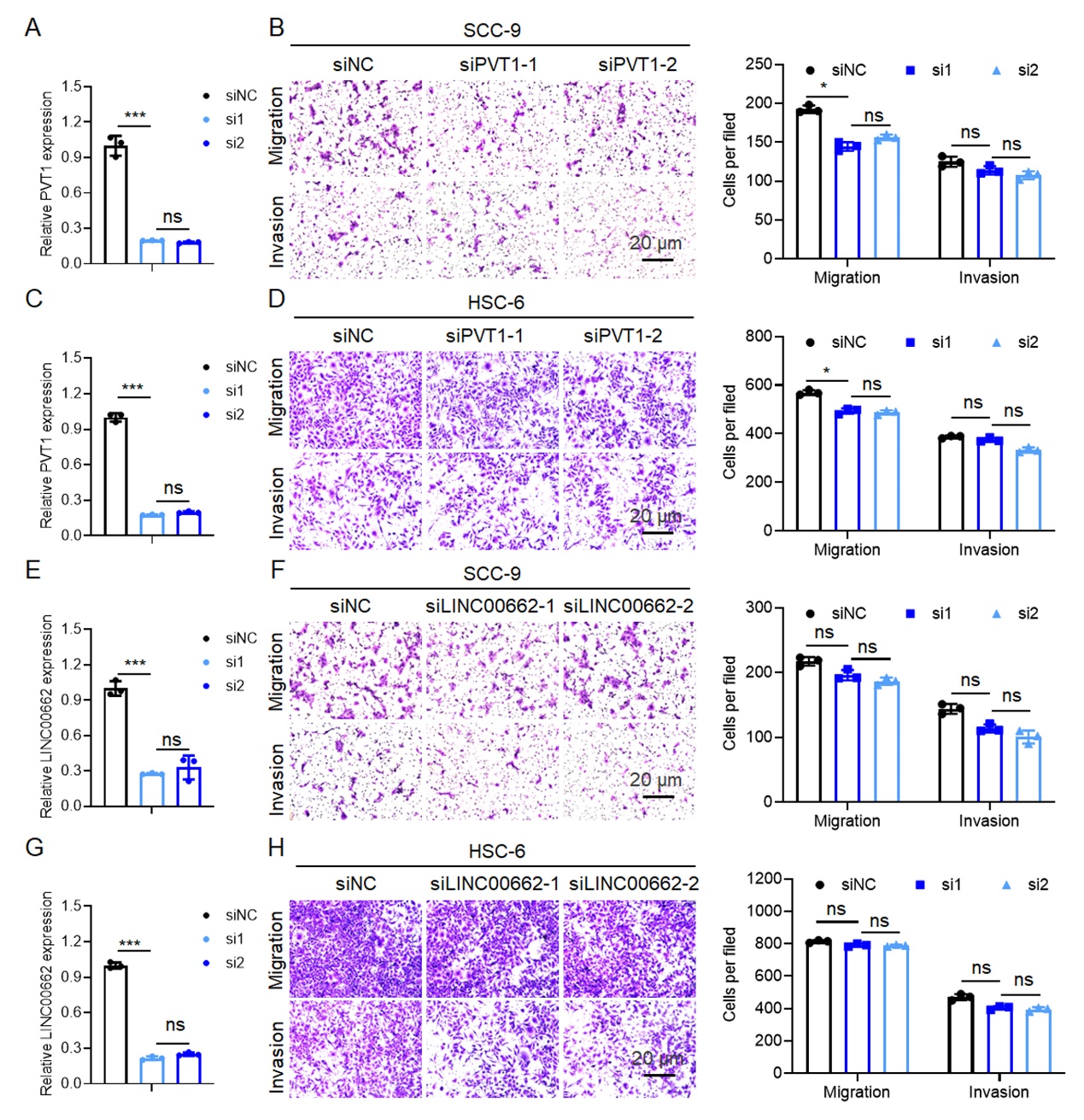


Figure S2

Knocking down lncRNAs and their effects on migration and invasion.

(A) Validation of PVT1 expression in SCC-9 cells with PVT1 knock down via qRT-PCR.

(B) Representative images and statistical analysis of transwell assays showing the migration and invasion abilities of SCC-9 cells after PVT1 was knocked down (scale bar: 20 μm).

(C) Validation of PVT1 expression in HSC-6 cells with PVT1 knock down via qRT-PCR.

(D) Representative images and statistical analysis of transwell assays showing the migration and invasion abilities of HSC-6 cells after PVT1 was knocked down (scale bar: 20 μm).

(E) Validation of LINC00662 expression in SCC-9 cells with LINC00662 knock down via qRT-PCR.

(F) Representative images and statistical analysis of transwell assays showing the migration and invasion abilities of SCC-9 cells after LINC00662 was knocked down (scale bar: 20 μm).

(G) Validation of LINC00662 expression in HSC-6 cells with LINC00662 knock down via qRT-PCR.

(H) Representative images and statistical analysis of transwell assays showing the migration and invasion abilities of HSC-6 cells after LINC00662 was knocked down (scale bar: 20 μm).

The error bars represent the SDs of independent experiments. * *p* < 0.05; ** *p* < 0.01; *** *p* < 0.001.


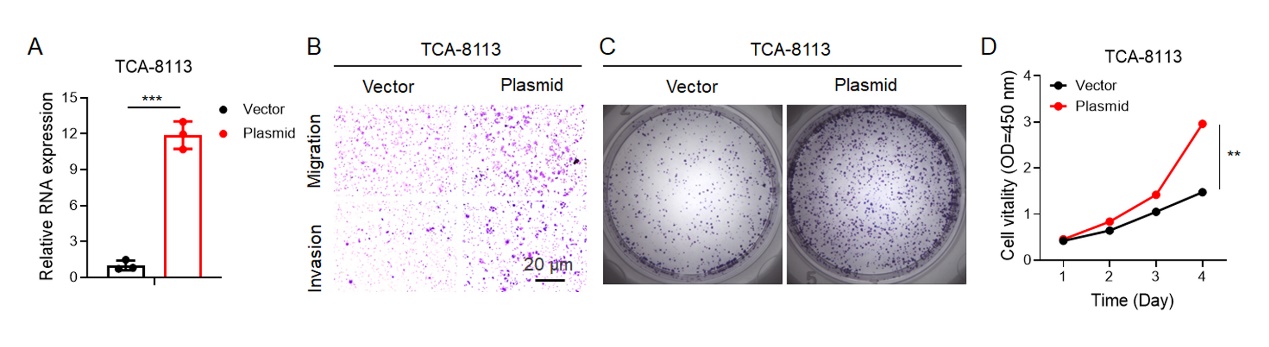


Figure S3

LncOCMRL1 overexpression promotes the invasion, migration and proliferation of OSCC cells.

(A) Validation of lncOCMRL1 expression in TCA-8113 cells with lncOCMRL1 overexpression by qRT-PCR.

(B) Representative images of migration and invasion after lncOCMRL1 was overexpressed in the TCA-8113 cells (scale bar: 20 μm).

(C) Representative images of the colony formation ability of TCA-8113 cells after lncOCMRL1 was overexpressed.

(D) Proliferation of the TCA-8113 cells after lncOCMRL1 was overexpressed.

The error bars represent the SDs of independent experiments. * *p* < 0.05; ** *p* < 0.01; *** *p* < 0.001.


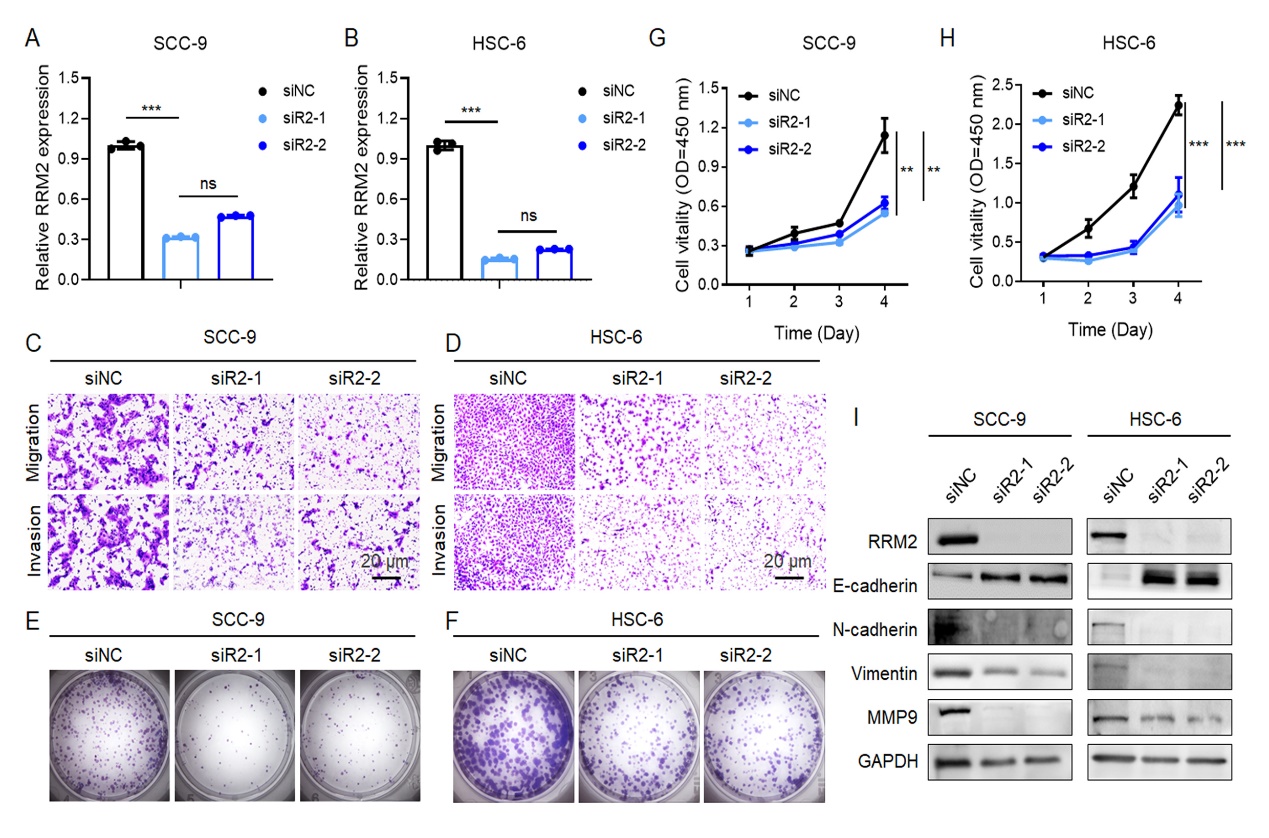


Figure S4

RRM2 silencing inhibits the invasion, migration, proliferation and EMT of OSCC cells.

(A-B) qRT-PCR analysis of RRM2 silencing efficiency in SCC-9 (A) and HSC-6 cells (B).

(C-D) Representative images of migration and invasion after RRM2 was silenced in SCC-9 (C) and HSC-6 cells (D) (scale bar: 20 μm).

(E-F) Representative images of colony formation after RRM2 was silenced in SCC-9 (E) and HSC-6 cells (F).

(G-H) Proliferation of SCC-9 (G) and HSC-6 cells (H) after RRM2 was knocked down.

(I) Western blotting analysis showing the effect of RRM2 silencing on EMT in SCC-9 and HSC-6 cells.

The error bars represent the SDs of independent experiments. * *p* < 0.05; ** *p* < 0.01; *** *p* < 0.001.


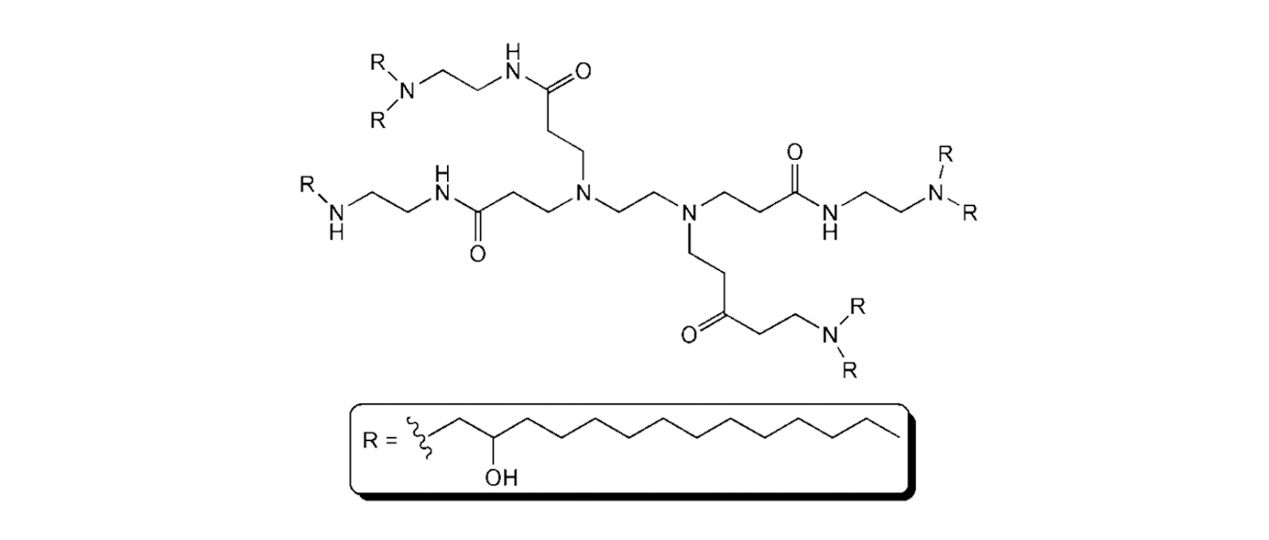


Figure S5

Chemical structure of the amphiphilic cationic lipid-like compounds G0-C14.


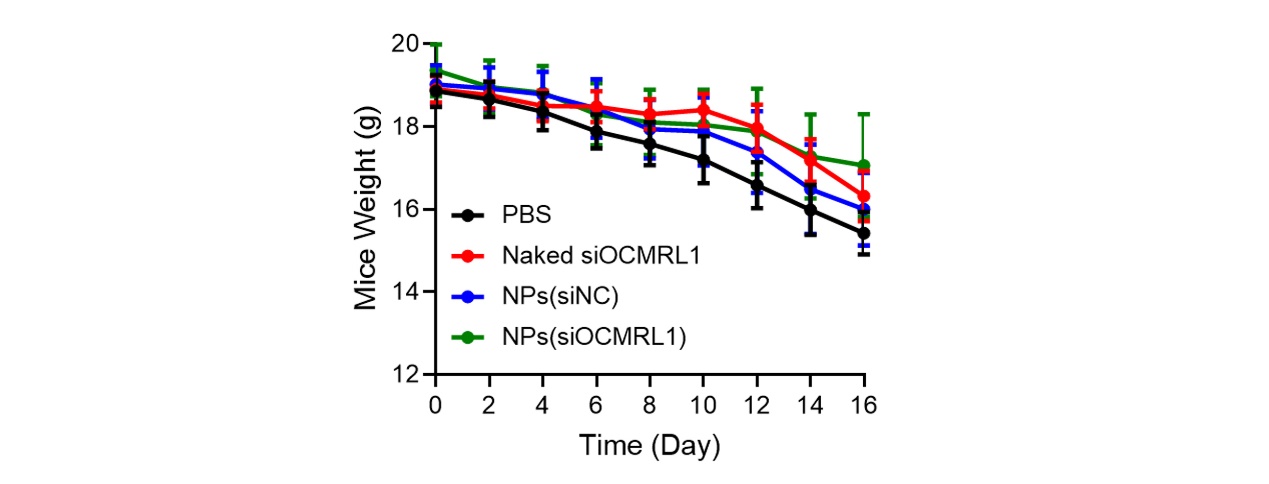


Figure S6

Statistical analysis of mouse body weight changes in the OSCC tongue orthotopic xenograft model.


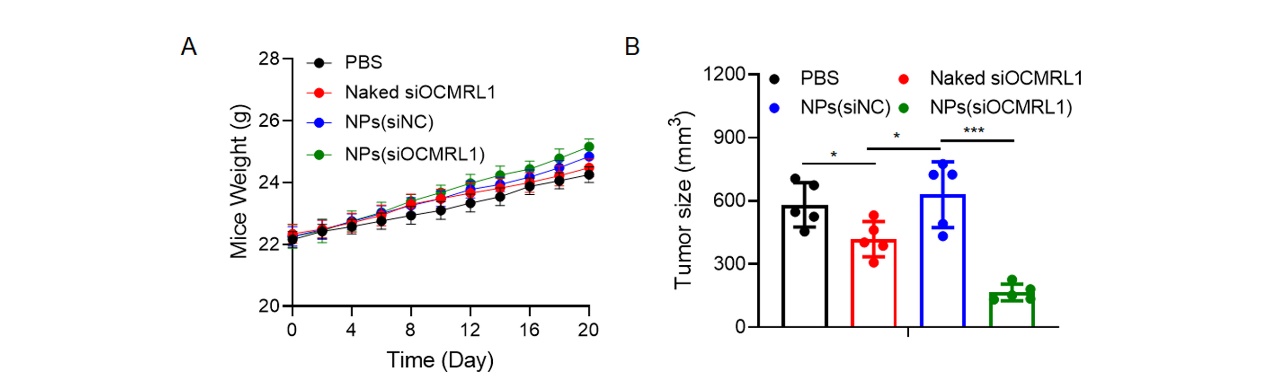


Figure S7

Mouse body weight changes and tumor volume in the PDX model.

(A) Statistical analysis of mouse body weight changes in the PDX model.

(B) Statistical analysis of tumor volume in the PDX model.

The error bars represent the SDs of independent experiments. * *p* < 0.05; ** *p* < 0.01; *** *p* < 0.001.


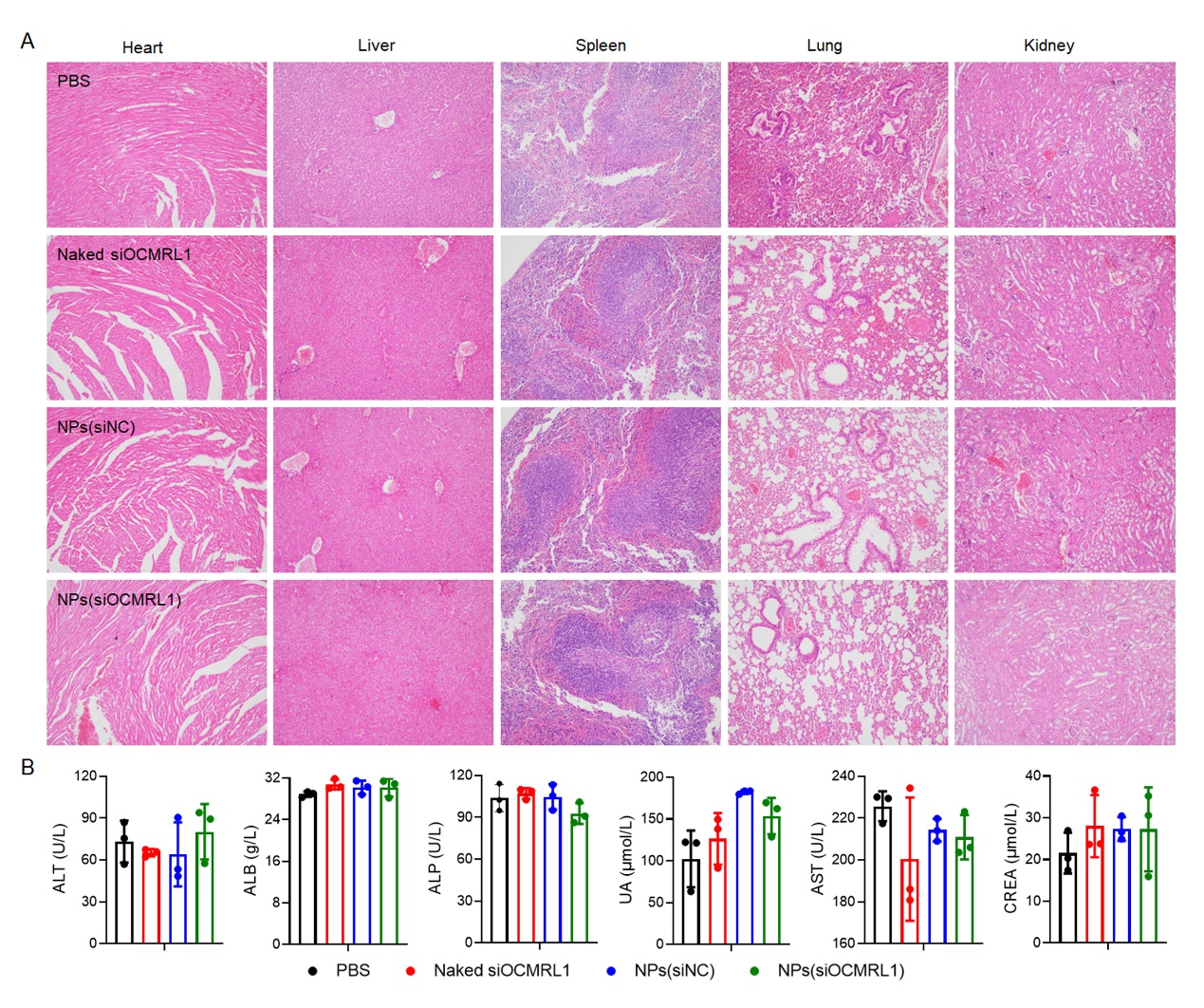


Figure S8

Biosafety of the NPs in OSCC tongue orthotopic xenograft model.

(A) HE staining of major organs of healthy mice (each group, n = 3) in the OSCC tongue orthotopic xenograft model.

(B) Hematological parameters, including alanine aminotransferase (ALT), albumin (ALB), alkaline phosphatase (ALP), uric acid (UA), aspartate aminotransferase (AST), and creatinine (CREA), were analyzed after treatment with different types of NPs and were within the normal range after treatment (n = 3) in the OSCC tongue orthotopic xenograft model.


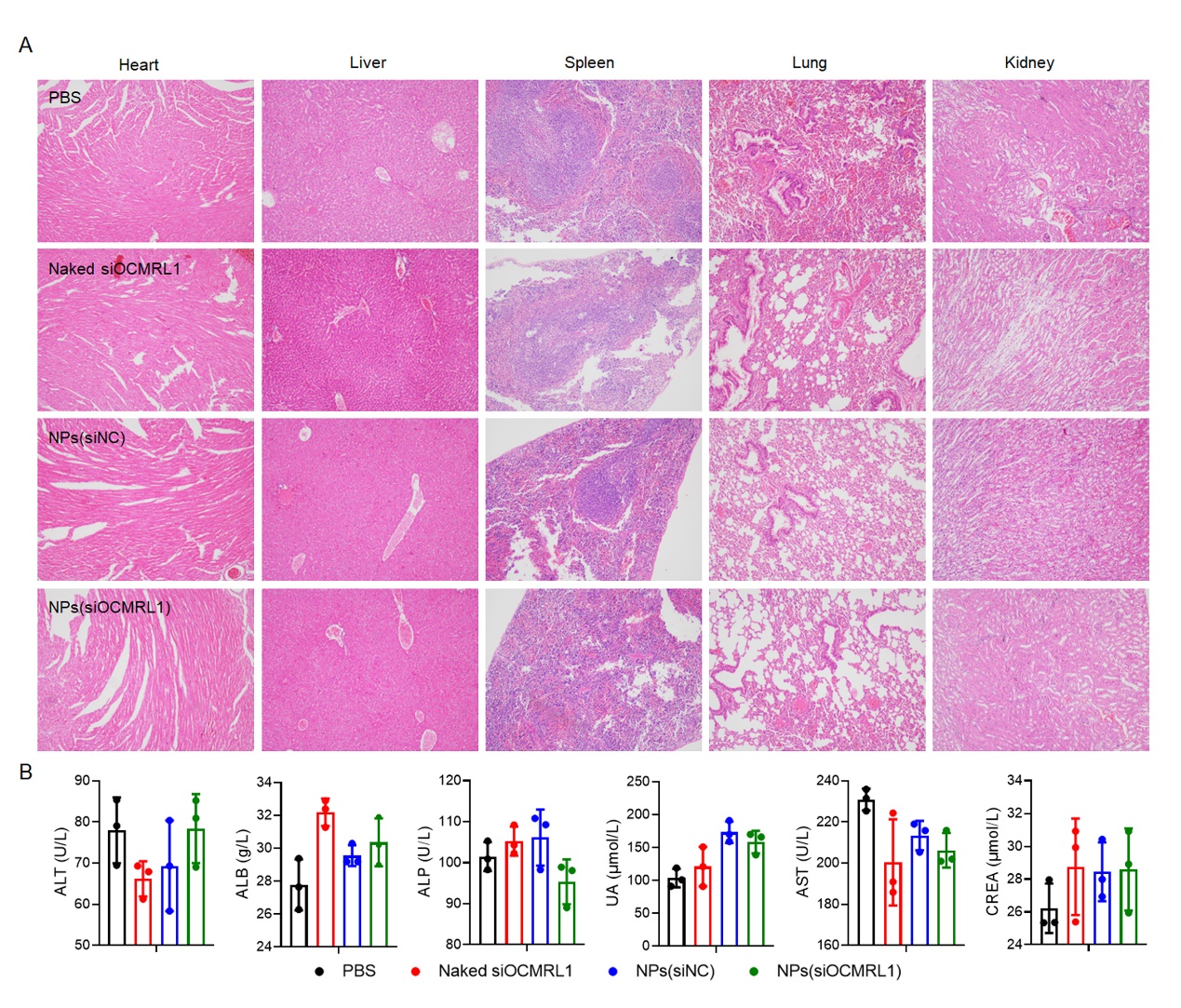


Figure S9

Biosafety of NPs in a tongue in the PDX model.

(A) HE staining of major organs of mice in the PDX model.

(B) Hematological parameters, including ALT, ALB, ALP, UA, AST, CREA, were analyzed after treatment with different types of NPs and were within the normal range after treatment (n = 3) in the PDX model.

**Table S1 Information on surgically resected tumor samples from patients without metastasis (n = 3) and patients with metastasis (n = 3).**

| Patient | Age | Sex | Differentiation | T stage | Lymphatic metastasis |
| --- | --- | --- | --- | --- | --- |
| #1 | 55 | Male | High | T2 | Yes |
| #2 | 71 | Male | Moderate | T2 | Yes |
| #3 | 46 | Female | High | T2 | Yes |
| #4 | 35 | Male | Moderate | T2 | No |
| #5 | 64 | Male | High | T2 | No |
| #6 | 51 | Male | High | T2 | No |
